# Multi-critical topological transition at quantum criticality

**DOI:** 10.1038/s41598-020-80337-7

**Published:** 2021-01-13

**Authors:** Ranjith R. Kumar, Y. R. Kartik, S. Rahul, Sujit Sarkar

**Affiliations:** 1 Department of Theoretical Sciences, Poornaprajna Institute of Scientific Research, 4, Sadashivanagar, Bangalore, 560 080 India; 2 Graduate Studies, Manipal Academy of Higher Education, Madhava Nagar, Manipal, 576104 India

**Keywords:** Superconducting properties and materials, Topological matter

## Abstract

The investigation and characterization of topological quantum phase transition between gapless phases is one of the recent interest of research in topological states of matter. We consider transverse field Ising model with three spin interaction in one dimension and observe a topological transition between gapless phases on one of the critical lines of this model. We study the distinct nature of these gapless phases and show that they belong to different universality classes. The topological invariant number (winding number) characterize different topological phases for the different regime of parameter space. We observe the evidence of two multi-critical points, one is topologically trivial and the other one is topologically active. Topological quantum phase transition between the gapless phases on the critical line occurs through the non-trivial multi-critical point in the Lifshitz universality class. We calculate and analyze the behavior of Wannier state correlation function close to the multi-critical point and confirm the topological transition between gapless phases. We show the breakdown of Lorentz invariance at this multi-critical point through the energy dispersion analysis. We also show that the scaling theories and curvature function renormalization group can also be effectively used to understand the topological quantum phase transitions between gapless phases. The model Hamiltonian which we study is more applicable for the system with gapless excitations, where the conventional concept of topological quantum phase transition fails.

## Introduction

Quantum phase transitions is one of the fascinating subject in condensed matter physics. Landau’s paradigm of spontaneous symmetry breaking describes continuous phase transitions successfully using local order parameter, which is finite at the ordered phase and vanishes at the critical point^1–4^. Contrary to this, topological quantum phase transitions (TQPT)—recently observed new class of phase transition—can be understood as a manifestation of topological properties of electronic band structure^5–7^, instead of local order parameter. There is no spontaneous symmetry breaking associated, and hence it is not possible to define local order parameter for the transition between topologically distinct gapped phases. Topological gapped phases are distinguished by quantized topological invariants, which takes discrete values across TQPT points^8,9^.

Despite the failure of Landau’s approach, recently, a theory of critical phenomena was found to be successful to extract the critical behavior and obtain universality classes by identifying critical exponents using scaling relations in TQPTs^10–14^. These TQPT points are essentially quantum critical points (QCP), since they occur at zero temperature. One can define spacial and temporal characteristic lengths that have diverging behavior as we approach QCP. This diverging property of characteristic lengths with critical exponent $\nu$ (correlation length exponent) and *z* (dynamical critical exponent), enable one to define universality classes of TQPTs^15–17^. Localized edge modes in the topological non-trivial phases tend to delocalize and penetrate into the bulk as one approaches the TQPT point. The exponential decay of edge modes into the bulk depends on the distance to the topological transition (*g*) and is characterized by a length scale $\xi =|g|^{-\nu }$. This characteristic length $\xi$ can be referred as correlation length with critical exponent $\nu$^18,19^. Correlation length exponent can be obtained using several approaches including the numerical studies of penetration length of the edge modes as a function of the distance to the transition^10,11^, and also from the scaling properties of the Berry connection^20–22^. At QCP energy dispersion $E_k$ is found to be $E_k\propto k^z$, where *z* is dynamical critical exponent. Expanding the energy dispersion around the QCP and identifying the dominant momentum one can find the value of *z*, which governs the shape of the spectra at the gap closing point^23^.

As one approaches TQPT point the system exhibits scale invariance. Exploiting this property, a scaling theory, analogous to the Kadanoff’s scaling theory of conventional critical phenomena^24^, has been proposed^20,25^. The topological invariant—calculated by integrating curvature function over the whole Brillouin zone in the momentum space—takes integer values for topological gapped phases and changes abruptly at the critical point. The curvature function diverges at the critical point signaling the critical behavior of TQPT point. Based on this behavior of curvature function a renormalization group (RG) approach has been developed^26,27^. A knot-tying scaling procedure is proposed based on the divergence in the curvature function at the critical point. This scaling procedure changes the curvature function and drives the system to its fixed point configuration, without changing the topology of the band structure. Since the topological invariant does not change during this process, the RG flow lines distinguish between distinct topological gapped phases. In one dimensional systems this scaling procedure is analogous to stretching a string until the knots are revealed^28^. This curvature function renormalization group (CRG) has been used in studying the topological phase transition in, Kitaev model, Su-Schrieffer–Heeger model^25^, periodically driven systems^27,29^, systems without inversion symmetry^30^, models with $Z_2$ invariant^31^, quantum walks that simulate one and two-dimensional Dirac models^32^, multi-critical 1D topological insulator^33^ and also in interacting systems^21,34^ etc.

All these characterizing tools mentioned above have been widely used to distinguish between gapped phases separated by a topological transition. However, the appearance of transition between stable gapless phases with trivial and non-trivial topological characters have also been observed in a wide class of magnetic systems^35–39^. Exponentially localized edge modes at the QCPs, in one and two-dimensional symmetry protected topological phases, are stable to disorder and can give rise to topologically distinct gapless phases^40,41^.

### Motivation

In this work, we are motivated to study the TQPT occurring between two gapless phases through a Lorentz symmetry breaking point. We consider transfer field Ising model (TFIM) with three spin interaction^42^, where the study of edge modes at criticality has revealed the appearance and disappearance of localized edge modes at one of the quantum critical lines with corresponding change in the parameter values^43^. In other words, both topological and non-topological characters appear on the same critical line for different parameter regimes. This provides an interesting platform to study TQPT between gapless phases as well as to understand the validity of characterizing tools in identifying this transition.

Motivation of this work is twofold. First is to prove that, indeed the critical line possess distinct gapless phases and there is a TQPT between these phases occurring through a multi-critical point which breaks the Lorentz invariance in our model Hamiltonian. Second one is to perform this using characterizing techniques that have been used to distinguish between gapped phases, thereby validating the reliability of these techniques to distinguish between gapless phases. We also show the relation between the breaking of Lorentz invariance and topological quantum phase transition at the multi-critical point. This phenomenon can be analogously understood from the topological semimetals, where the Dirac points confluence to form quadratic dispersion at a critical point which breaks the Lorentz symmetry^44,45^.

There are several studies on multi-critical behavior and topological transition using conventional RG techniques in the literature^46–50^. The conventional RG captures the physics of correlated topological systems with local Coulomb interaction in one, two and three dimensions. However, here we adopt CRG based on the diverging behavior of curvature function as we approach the topological quantum critical point. Since the curvature function encapsulates the topological signatures of the band structure, its prominent behavior near the transition point is promising and sufficient to address the unconventional topological transition between gapless phases in our model.

## Model hamiltonian and topological quantum phase diagram

We consider transverse field Ising model with three spin interaction^42,51^

1 $$\begin{aligned} H=-\sum _{i} \left( \lambda _1 \sigma _{i}^z \sigma _{i-1}^z + \lambda _2 \sigma _{i}^x \sigma _{i-1}^z \sigma _{i+1}^z + \mu \sigma _{i}^x\right) , \end{aligned}$$

where $\sigma ^{x,z}$ are Pauli matrices. Performing Jordan–Wigner transformation $\sigma _{i}^x=1-2c_i^{\dagger }c_i$ and $\sigma _{i}^z= - \prod _{j<i} (1-2c_{j}^{\dagger }c_{j}) (c_{i} + c_{i}^{\dagger })$, the model Hamiltonian can be written in spinless fermionic form as

2 $$\begin{aligned} H = -\mu \sum _{i=1}^{N} (1 - 2 c_{i}^{\dagger }c_{i}) - \lambda _1 \sum _{i=1}^{N-1} (c_{i}^{\dagger }c_{i+1} + c_{i}^{\dagger }c_{i+1}^{\dagger } + h.c) - \lambda _2 \sum _{i=2}^{N-1} ( c_{i-1}^{\dagger }c_{i+1} + c_{i+1} c_{i-1} + h.c), \end{aligned}$$

where nearest neighbor superconducting gap is equal to nearest neighbor hopping amplitude $\lambda _1$ and next nearest neighbor superconducting gap is equal to next nearest neighbor hopping amplitude $\lambda _2$. In this equation, $c_i^{\dagger }(c_i)$ is creation (annihilation) fermionic operator and *h*.*c* represents the Hermitian conjugate. It is a one-dimensional mean-field model for a triplet superconductor. The three spin interaction added to the transverse field Ising model can be physically realized in realistic Hamiltonians since the term is generated through real-space renormalization group treatments^42^.

The Bloch Hamiltonian of Eq. (2), which is a $2\times 2$ matrix, can be written as

3 $$\begin{aligned} \mathscr {H}(k) = \chi _{z} (k) \sigma _z - \chi _{y} (k) \sigma _y , \end{aligned}$$

where $\chi _{z} (k) = -2 \lambda _1 \cos k - 2 \lambda _2 \cos 2k + 2\mu ,$ and $\chi _{y} (k) = 2 \lambda _1 \sin k + 2 \lambda _2 \sin 2k.$ The excitation spectra can be obtained as

4 $$\begin{aligned} E_k=\pm \sqrt{\chi _{z}^2 (k) + \chi _{y}^2 (k)}. \end{aligned}$$

**Figure 1:**
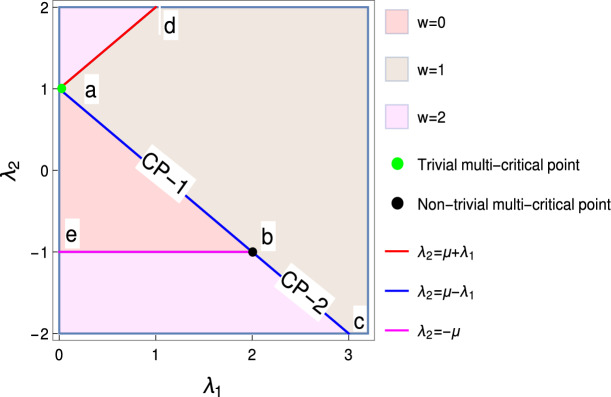
Topological phase diagram of model Hamiltonian for $\mu =1$. Line ‘ac’ represents the critical line $\lambda _2=\mu -\lambda _1$ (blue line), line ‘be’ represents the critical line $\lambda _2=-\mu$ (magenta line) and line ‘ad’ represents the critical line $\lambda _2=\mu +\lambda _1$ (red line). Points ‘a’ and ‘b’ are multi-critical points (green and black dots respectively) which differentiate between three distinct gapped phases with $w=0,1,2$ (represented in different colors). Here CP-1 is critical/gapless phase for the transition between $w=0$ and $w=1$. CP-2 is critical/gapless phase for the transition between $w=1$ and $w=2$.

This model supports topological distinct gapped phases (i.e $w=0,1,2$) separated by the three quantum critical lines as shown in Fig. 1. The energy gap closes at these quantum critical lines, $\lambda _2=\mu +\lambda _1$, $\lambda _2=\mu -\lambda _1$ and $\lambda _2=-\mu$, obtained for momentum $k_0=\pm \pi$, $k_0=0$ and $k_0=\cos ^{-1}(-\lambda _1/2\lambda _2)$ respectively. The topological angle can be written as $\phi _k=\tan ^{-1}\left( \chi _{y} (k)/\chi _{z} (k) \right)$.

The model has been studied previously in different contexts^42,43,51,52^. The model was first introduced by the authors of Ref.^42^ to study the persistence of quantum criticality at high temperature in correlated systems. The authors of Ref.^52^ has studied the physics of Majorana zero modes in the gapped phases of this model with both broken and unbroken time-reversal symmetry. One of the authors (S.S) has studied the quantization of geometric phase with integer and fractional topological characterization for this model in Ref.^51^. Very recently authors of Ref.^43^ have solved the problem of bulk-boundary correspondence at the quantum critical lines and discussed the principle of least topological invariant number at the criticality.

In this work we intent to show explicitly that there exist a TQPT between two gapless phases (CP-1 and CP-2 in Fig. 1) on the critical line $\lambda _2=\mu -\lambda _1$ through a multi-critical point $\lambda _1=2\mu$ (point ‘b’ in Fig. 1). We also explore the nature of transition and critical behavior implementing the scaling law of critical theories and show that these characterizing tools, which are used to characterize the transition between gapped phases, are also efficient tools to characterize the TQPT between gapless phases.

There are two multi-critical points at the intersections of the critical lines. For the parameter value $\mu =1$ a multi-critical point with an emergent *U*(1) symmetry exist at $(\lambda _1,\lambda _2)=(0,1)$^52^. This multi-critical point ‘a’ in the phase diagram (Fig. 1) occurs at the intersection of the critical lines $\lambda _2=\mu +\lambda _1$ and $\lambda _2=\mu -\lambda _1$. It posses linear spectra at the gap closing momenta $k=0$ and $k=\pm \pi$ and does not break the Lorentz invariant. Since it does not involve any topological transition between gapless phases on a critical line, we consider it a trivial multi-critical point. Another multi-critical point exist at $(\lambda _1,\lambda _2)=(2,-1)$. This multi-critical point ‘b’ in the phase diagram occurs at the intersection of critical lines $\lambda _2=\mu -\lambda _1$ and $\lambda _2=-\mu$. Since it posses quadratic spectra at $k=0$ and breaks Lorentz invariance, we consider it to be a non-trivial multi-critical point. This is exactly the point $\lambda _1=2\mu$, through which TQPT between gapless phases occur.

**Figure 2:**
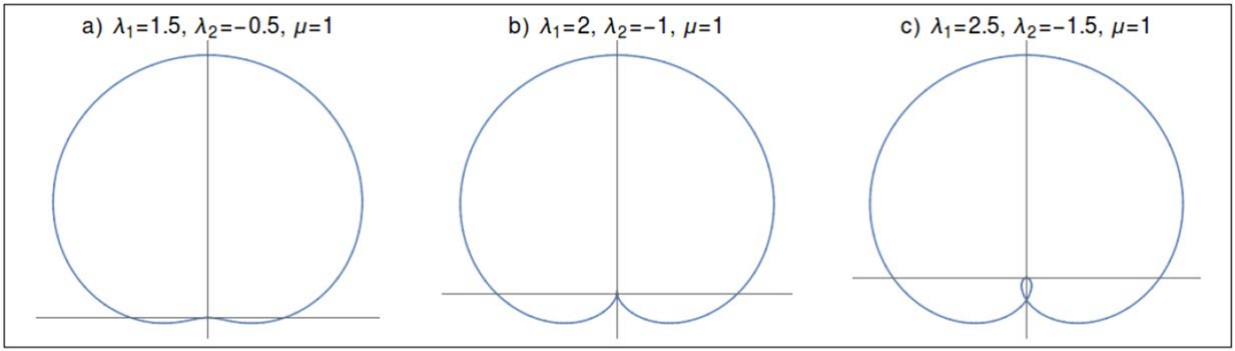
Parameter space for pseudo spin-vector on the critical line $\lambda _2=\mu -\lambda _1$. (**a**) Trivial gapless phase (**b**) multi-critical point (**c**) non-trivial gapless phase.

The transition can be verified by investigating behavior of pseudo spin-vector in the parameter space^51,53^. The model Hamiltonian can be expressed in terms of pseudo spin-vector as

5 $$\begin{aligned} \mathscr {H}(k)= \varvec{\chi }(k).\varvec{\sigma }, \end{aligned}$$

where $\chi _{z} (k) = -2 \lambda _1 \cos k - 2 \lambda _2 \cos 2k + 2\mu ,$ and $\chi _{y} (k) = 2 \lambda _1 \sin k + 2 \lambda _2 \sin 2k$. The pseudo spin-vector takes a closed curve in the parameter space around the origin for a set of parameter values representing a gapped phase. For gapless phase the curve passes through the origin and this behavior is characteristic of criticality. In Fig. 2 we have shown the behavior of pseudo spin-vector in the parameter space on the critical line $\lambda _2=\mu -\lambda _1$. The curve is always closed and passes through the origin indicating the criticality. As one goes from Fig. 2a–c, system is passing from topologically trivial gapless phase to non-trivial gapless phase through a multi-critical point (Fig. 2b). Trivial gapless phase is the phase boundary between $w=0$ and $w=1$ gapped phases, as well as, non-trivial gapless phase is the phase boundary between $w=1$ and $w=2$ gapped phases. The non-trivial gapless phase is characterized by the emergence of secondary loop which passes through the origin. Therefore this behavior of pseudo spin-vector suggest that there exist a TQPT between two gapless phases on the critical line $\lambda _2=\mu -\lambda _1$.

## Results and discussion

### Energy dispersion and critical exponents

One can distinguish between the universality classes of the gapless phases by calculating the values of critical exponents. In this section we calculate the correlation length critical exponent ($\nu$) and dynamical critical exponent (*z*) for the two gapless phases on the critical line $\lambda _2=\mu -\lambda _1$.

**Figure 3:**
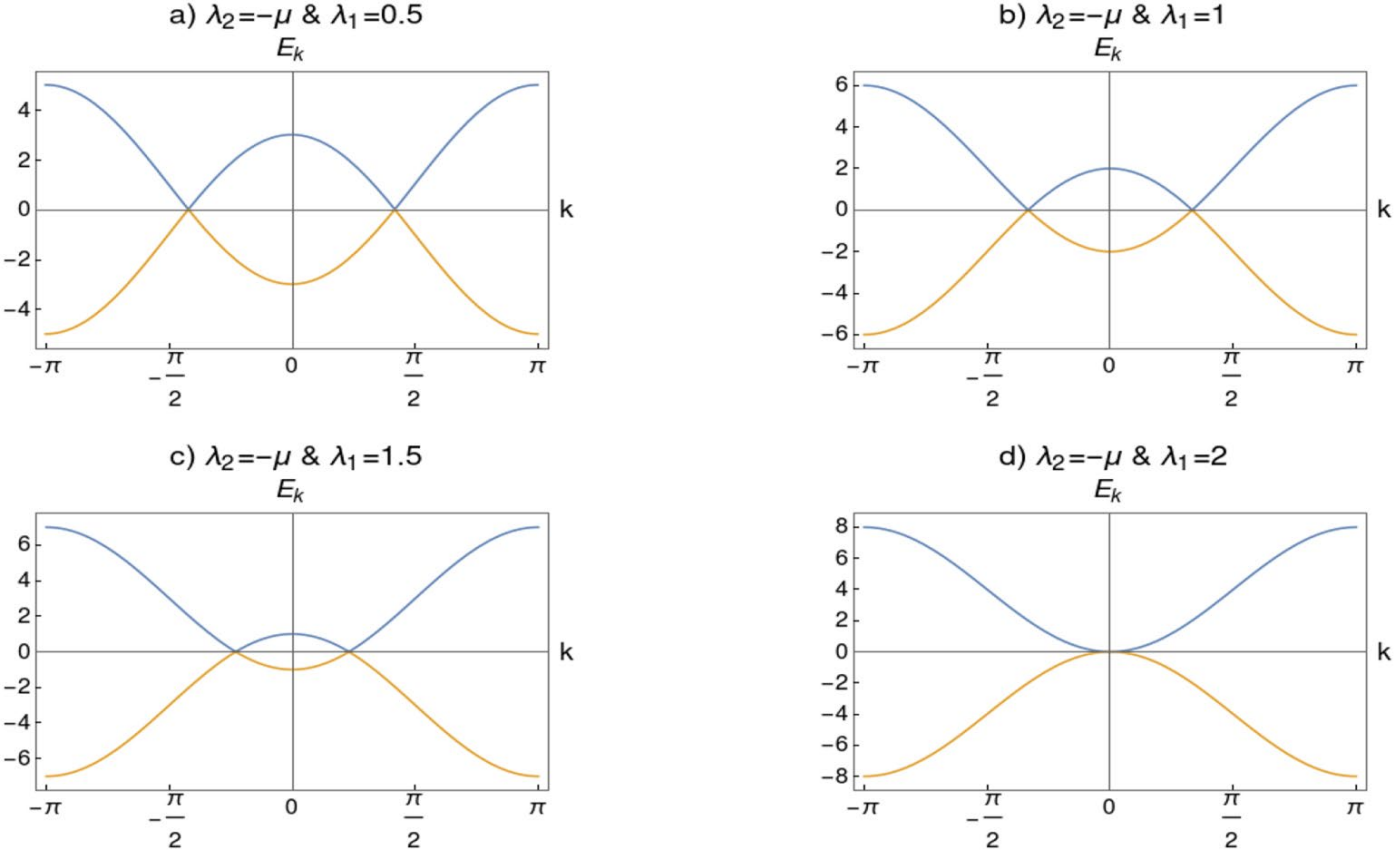
(**a**–**c**) Spectra on the critical line $\lambda _2=-\mu$ (with $\mu =1$). There are two gapless points around which the spectra is linear (i.e., $E_k\propto k$) which implies $z=1$. (**d**) Spectra at multi-critical point with $\lambda _1=2\mu$ and $\lambda _2=-\mu$. Two gapless points confluence at $k=0$ where the spectra is quadratic (i.e., $E_k\propto k^2$) and $z=2$.

The spectra of this model on the critical line $\lambda _2=\mu -\lambda _1$ is gapless and linear for $\lambda _1<2\mu$, and quadratic for $\lambda _1\ge 2\mu$. On the critical line $\lambda _2=-\mu$ spectra has two gapless points at the two incommensurate momenta, $\pm k_0$, symmetric about the point $k=0$ as shown in Fig. 3a–c. As we approach multi-critical point on this critical line, the two incommensurate points confluence at $k_0=0$ (i.e, $(\lambda _1,\lambda _2)=(2,-1)$), as shown in Fig. 3d. Therefore the spectra is non-relativistic (breaks Lorentz invariance) and become quadratic in nature instead of linear. Energy dispersion for one dimensional system close quantum critical point can be written as $E_k=\sqrt{|g|^{2\nu z}+k^{2z}}$, where $\nu$ is correlation length critical exponent and *z* is dynamical critical exponent^23^. At the critical point the gap function $\Delta =|g|^{2\nu z}$ should go to zero, therefore $E\propto k^z$.

The energy dispersion expanded around the gap closing momenta $k_0 = 0$ can be written as

6 $$\begin{aligned} E_k=\pm \sqrt{(2\mu -2\lambda _1-2\lambda _2)^2 + C_2 k^2 +C_4 k^4}, \end{aligned}$$

where $C_2= (16\lambda _2\mu +4\lambda _1\mu -4\lambda _1\lambda _2)$ and $C_4= \frac{1}{3}(\lambda _1\lambda _2-\lambda _1\mu -16\lambda _2\mu )$. Gap function $g^{2\nu z}=(2\mu -2\lambda _1-2\lambda _2)^2$ implies $\nu z =1$. At QCP the gap function goes to zero and the shape of the spectra can be obtained as $E_k \propto k^z$, by identifying the dominant coefficient among $C_2$ and $C_4$. Above the multi-critical point (trivial gapless phase, i.e., $\lambda _1<2\mu$) one can observe that the coefficient of quadratic term $C_2$ is much larger than $C_4$. Therefore quadratic term dominate implying $E_k\propto k$, hence $z=1$. Similarly below the multi-critical point (non-trivial gapless phase, i.e., $\lambda _1>2\mu$) one can find that $C_4$ dominates over $C_2$ and the spectra $E_k \propto k^2$ implying the value of $z=2$. At the multi-critical point (i.e, $\lambda _1=2\mu$ and $\lambda _2=-\mu$) the coefficient $C_2=0$, which entails $z=2$ since $E_k\propto k^2$. Therefore the dynamical critical exponent is found to have $z=1$ with linear spectra at the trivial gapless phase and $z=2$ with quadratic spectra at transition point (multi-critical point) as well as non-trivial gapless phase. Once the dynamical critical exponent *z* is obtained one can also obtain the value of correlation length critical exponent $\nu$ from the condition $\nu z=1$ in our model. Thus in the trivial gapless phase the critical exponents are $z=1$ and $\nu =1$ and in the non-trivial gapless phase $z=2$ and $\nu =\frac{1}{2}$. Note that the situation $C_2=C_4$ is not possible on the critical line since it requires $\lambda _1$ to be complex. Equating $C_2$ and $C_4$ results in $\lambda _1\propto \left( 4 \mu -i \sqrt{177} \mu \right)$, which is not possible in our model, implying $C_2\ne C_4$.

This observation suggest that these two gapless phases belong to different universality classes since their critical exponents has different set of values. This entails the fact that there is a TQPT in the Lifshitz universality class with $z=2$ and $\nu =\frac{1}{2}$^23,54,55^, between two distinct gapless phases through multi-critical point. Thus in this study the breaking of Lorenz invariance occurs at the Lifshitz universality class.

**Figure 4:**
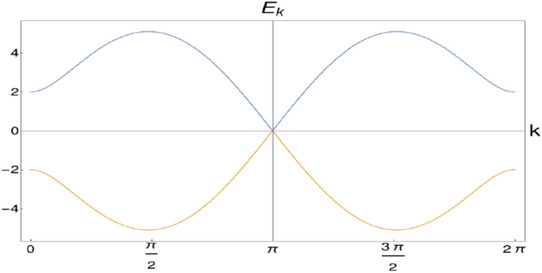
Spectra on the critical line $\lambda _2=\mu +\lambda _1$.

For completeness we also calculate the critical exponents for the critical theory at $\lambda _2=\mu +\lambda _1$. The spectra on this line is found to be linear in *k* as shown in Fig. 4, which implies the value of dynamical critical exponent to be $z=1$. Spectra close to $k_0=\pm \pi$ can be written as

7 $$\begin{aligned} E_k = \pm \sqrt{(2\mu +2\lambda _1-2\lambda _2)^2+C_2 k^2 + C_4 k^4}, \end{aligned}$$

where $C_2=(4\lambda _1\lambda _2-4\lambda _1\mu +4\lambda _2\mu )$ and $C_4=\frac{1}{3}(\lambda _1\mu -\lambda _1\lambda _2-16\lambda _2\mu )$. At the QCP gap function goes to zero and coefficient $C_2$ dominates over $C_4$, implying $E_k \propto k$. Therefore the spectra at the gap closing point is linear and dynamical critical exponent $z=1$. The gap function $g^{2\nu z}= (2\mu +2\lambda _1-2\lambda _2)^2$ implies $\nu =1$.

We have shown the breakdown of Lorentz invariant symmetry at the multi-critical point. The authors of Ref.^56–58^ have shown explicitly that the break down of Lorentz invariance also occur for graphene and 3D Weyl semimetal. The authors of Ref.^59^ have shown explicitly the transformation from the Dirac semimetal to band insulator QCP at $\Delta =0$, ($\Delta$ is the energy scale), where the quasiparticle spectra is two momentum space dimension. In *x*-direction, it is linear in *k* and in the *y*-direction it is quadratic ($k^2$). But the model Hamiltonian which we have studied is one dimension, therefore only one component has appeared.

We confirm the results for our model by calculating the critical exponents from the Berry connection approach and also show the presence of TQPT between gapless phases using CRG analysis in the next section.

### Curvature function renormalization group

At first, we briefly review the curvature function renormalization group (CRG) method which encapsulates the critical behavior of a system during topological phase transition. Let us consider a system with a set of parameters $\mathbf {M}=(M_1,M_2,M_3, \ldots )$, which upon tuning appropriately changes the underlying topology of the system and induces topological phase transition. The curvature function $F(k,\mathbf {M})$ at momentum *k* dictate the topological properties of the system. Integral of this curvature function over a Brillouin zone defines topological invariant number which characterizes a gapped phase. For 1D systems it reads

8 $$\begin{aligned} w=\int \limits _{-\pi }^{\pi }\frac{dk}{2\pi } F(k,\mathbf {M}). \end{aligned}$$

Change in this topological invariant number involves the phase transition between the distinct gapped phases. For 1D systems Berry connection is the curvature function. Since Berry connection is gauge dependent, one can choose the gauge for which $F(k,\mathbf {M})$ can be written in Ornstein-Zernike form around the high symmetry point (HSP) $k_0$,

9 $$\begin{aligned} F(k_0+\delta k,\mathbf {M})=\frac{F(k_0,\mathbf {M})}{1\pm \xi ^2\delta k^2}, \end{aligned}$$

where $\delta k$ is small deviation from HSP, and $\xi$ is characteristic length scale. As the system approaches critical point to undergo topological phase transition i.e, $\mathbf {M}\rightarrow \mathbf {M}_c$, curvature function diverges and changes sign as system moves across critical point

10 $$\begin{aligned} \lim _{\mathbf {M}\rightarrow \mathbf {M}_c^+}F(k_0,\mathbf {M})= -\lim _{\mathbf {M}\rightarrow \mathbf {M}_c^-}F(k_0,\mathbf {M})=\pm \infty . \end{aligned}$$

Based on the divergence of the curvature function near HSPs, a scaling theory has been developed. For given $\mathbf {M}$ we find new $\mathbf {M}^{\prime }$ which satisfies

11 $$\begin{aligned} F(k_0,\mathbf {M}^{\prime })=F(k_0+\delta k,\mathbf {M}), \end{aligned}$$

where $\delta k$ satisfies $F(k_0+\delta k,\mathbf {M})=F(k_0-\delta k,\mathbf {M})$. If the topology of the system at $\mathbf {M}$ and at fixed point $\mathbf {M}_f$ are same then the curvature function can be written as $F(k,\mathbf {M})=F_f(k,\mathbf {M}_f)+F_d(k,\mathbf {M}_d)$, where $F_f(k,\mathbf {M}_f)$ is curvature function at fixed point and $F_d(k,\mathbf {M}_d)$ is deviation from the fixed point. Applying Eq. (11) iteratively makes $F_d(k,\mathbf {M}_d) \rightarrow 0$, implying gradual decrease in the deviation of curvature function from the fixed point configuration. Hence $F(k,\mathbf {M})\rightarrow F_f(k,\mathbf {M}_f)$. Finding the map from $\mathbf {M}$ to $\mathbf {M}^{\prime }$ iteratively, broadens the curvature function $F(k_0, \mathbf {M})$ until it reaches fixed point. This iterative procedure yields RG flow in parameter space indicating critical points of the system. Generic RG equation of parameters $\mathbf {M}$ can be obtained by expanding Eq. (11) to leading order and writing $d\mathbf {M}=\mathbf {M}^{\prime }-\mathbf {M}$ and $\delta k^2=dl$, as^25,26^

12 $$\begin{aligned} \frac{d\mathbf {M}}{dl}= \frac{1}{2} \frac{\partial _k^2 F(k,\mathbf {M})|_{k=k_0}}{\partial _{\mathbf {M}}F(k_0,\mathbf {M})}. \end{aligned}$$

The critical point can be defined by the condition $|\frac{d\mathbf {M}}{dl}|=\infty$, and fixed point can be defined by the condition $|\frac{d\mathbf {M}}{dl}|=0$. As we approach critical point, along with the divergence of the curvature function [Eq. (10)], characteristic length $\xi$ in Eq. (9) also diverges

$$\begin{aligned} \lim _{\mathbf {M}\rightarrow \mathbf {M}_c}\xi =\infty . \end{aligned}$$

These divergences in $F(k_0,\mathbf {M})$ and $\xi$ give rise to divergent behavior characterized by the critical exponents

13 $$\begin{aligned} F(k_0,\mathbf {M}) \propto |\mathbf {M}-\mathbf {M}_c|^{-\gamma } \;\;\;,\;\;\;\;\;\; \xi \propto |\mathbf {M}-\mathbf {M}_c|^{-\nu }. \end{aligned}$$

In conventional Landau theory of phase transition with order parameter, correlation function plays prime role. The same can not be defined for topological phase transitions since there is no local order parameter. However, a correlation function in terms of a matrix element between Wannier states of distant home cells is proposed to characterize the topological phase transition^20^. This Wannier state correlation function $\lambda _R$, can be obtained from Fourier transform of the curvature function for 1D systems as

14 $$\begin{aligned} \lambda _R = \int \limits \frac{dk}{2\pi } e^{ikR}F(k,\mathbf {M}). \end{aligned}$$

Substituting the Ornstein–Zernike form of curvature function yields $\lambda _R\propto e^{-\frac{R}{\xi }}$. This suggest that $\xi$ can be treated as correlation length of topological phase transition with critical exponent $\nu$. Similarly curvature function at HSP, $F(k_0,\mathbf {M})$ has the notion of susceptibility in the Landau paradigm with the critical exponent $\gamma$. These critical exponents define the universality class of a model undergoing topological phase transition. A generic scaling law—imposed by the conservation of topological invariant—can be deduced for the critical exponents as

15 $$\begin{aligned} \gamma =\sum \limits _{i=1}^{D} \nu _i, \end{aligned}$$

where *D* is the dimensionality of the system. Thus for 1D systems we have $\gamma =\nu$^20^. The CRG method has been used to understand topological transition between gapped phases. Here we use this method to understand the topological transition between previously discussed gapless phases in our model. We calculate the RG equations and critical exponents for the critical theories between both gapped and gapless transitions and ensure the reliability of this method.

#### CRG for the transition between gapped phases

In this section we perform CRG for the topological transition across the critical line $\lambda _2=\mu -\lambda _1$, i.e, between the gapped phases with $w=0,1$ and 2. The objective of this discussion is to distinguish between the distinct critical phases CP-1 and CP-2 . We derive RG equations to confirm the topological transition between the gapped phases (between $w=0,2$ and $w=1$). We derive critical exponents for the CP-1 and CP-2 through Berry connection approach^23^ to characterize their universality classes. Transition between the CP-1 and CP-2 through the multi-critical point ‘b’ is studied in the next section.

**Figure 5:**
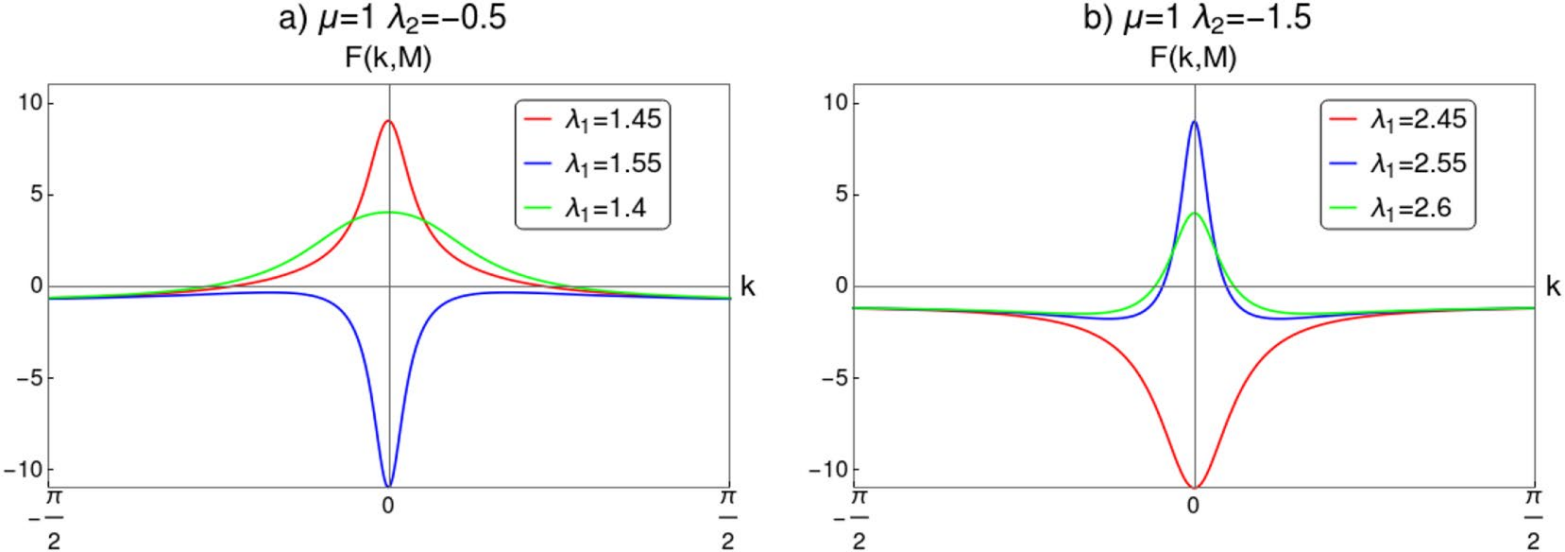
Curvature function $F(k,\mathbf {M})$ near the HSP $k_0=0$ plotted for $\lambda _2<0$. (**a**) Curvature function plotted for several values of $\lambda _1$ at $\mu =1$ and $\lambda _2=-0.5$ for the transition between $w=0$ and $w=1$. (**b**) Curvature function plotted for several values of $\lambda _1$ at $\mu =1$ and $\lambda _2=-1.5$ for the transition between $w=2$ and $w=1$. In both (**a**) and (**b**) the plot is around the QCPs, which defines the topological transition between gapped phases. As the QCP is approached, curvature function diverges at HSP and flips sign as we cross it. The scaling procedure proposed in CRG will fit here since the condition $F(k_0,\mathbf {M}^{\prime })=F(k_0+\delta k,\mathbf {M})$ is satisfied.

The curvature function can be calculated as

16 $$\begin{aligned} \begin{aligned} F(k,\mathbf {M})&= \dfrac{d\phi _k}{dk}\\&= \dfrac{d}{dk}\left[ \tan ^{-1}\left( \frac{2 \lambda _2 \sin (2 k) +2 \lambda _1 \sin (k)}{2 \mu -2 \lambda _2 \cos (2 k) -2 \lambda _1 \cos (k)}\right) \right] \\&= \frac{\lambda _1 \cos (k) (\mu -3 \lambda _2)+2 \lambda _2 \mu \cos (2 k)-\lambda _1^2-2 \lambda _2^2}{2 \lambda _1 \cos (k) (\lambda _2-\mu )-2 \lambda _2 \mu \cos (2 k)+\lambda _1^2+\lambda _2^2+\mu ^2}, \end{aligned} \end{aligned}$$

where $\mathbf {M}= \left\{ \mu , \lambda _1, \lambda _2\right\}$. Behavior of $F(k,\mathbf {M})$ near the QCPs for the transition between gapped phases is shown in Fig. 5. The transition between $w=0$ and $w=1$ is shown in Fig. 5a for the parameter values $\lambda _2=-0.5$ and $\mu =1$. For this transition critical point is obtained for $\lambda _1=1.5$ at $k_0=0$. In Fig. 5b, curvature function for transition between $w=2$ and $w=1$ for parameter values $\lambda _2=-1.5$ and $\mu =1$ is shown, where the critical point appear for $\lambda _1=2.5$ at $k_0=0$. Curvature function tend to diverge as we approach the critical points and flips sign as we cross it. This confirms that $F(k,\mathbf {M})$ takes the Ornstein–Zernike form of Eq. (9) around the HSP $k_0=0$. RG flow equations can be constructed now to see the flow line’s behavior in the parameter space to understand the topological transition in the model. The RG equations can be derived for $k_0=0$ as (refer to “Method” section for a detailed derivation)

17 $$\begin{aligned} \dfrac{d\lambda _1}{dl}= & {} \frac{{\lambda _1 }^2+{\lambda _1} (\mu -{\lambda _2 })+8 {\lambda _2 } \mu }{2 ({\lambda _1 }+{\lambda _2 }-\mu )},, \end{aligned}$$

18 $$\begin{aligned} \dfrac{d\lambda _2}{dl}= & {} -\frac{({\lambda _2 }+\mu ) \left( {\lambda _1 }^2+{\lambda _1 } (\mu -{\lambda _2 })+8 {\lambda _2 } \mu \right) }{2 ({\lambda _1 }-2 \mu ) ({\lambda _1 }+{\lambda _2 }-\mu )}, , \end{aligned}$$

19 $$\begin{aligned} \dfrac{d\mu }{dl}= & {} -\frac{({\lambda _2 }+\mu ) \left( {\lambda _1 }^2+{\lambda _1 } (\mu -{\lambda _2 })+8 {\lambda _2 } \mu \right) }{2 ({\lambda _1 }+2 {\lambda _2 }) ({\lambda _1 }+{\lambda _2 }-\mu )}. . \end{aligned}$$

For a constant value of $\mu$, Eqs. (17) and (18) satisfy the conditions

20 $$\begin{aligned} \left| \dfrac{d\lambda _1}{dl}\right| =\left| \dfrac{d\lambda _2}{dl}\right| = \infty \quad \text {and} \quad \left| \dfrac{d\lambda _1}{dl}\right| =\left| \dfrac{d\lambda _2}{dl}\right| = 0. \end{aligned}$$

**Figure 6:**
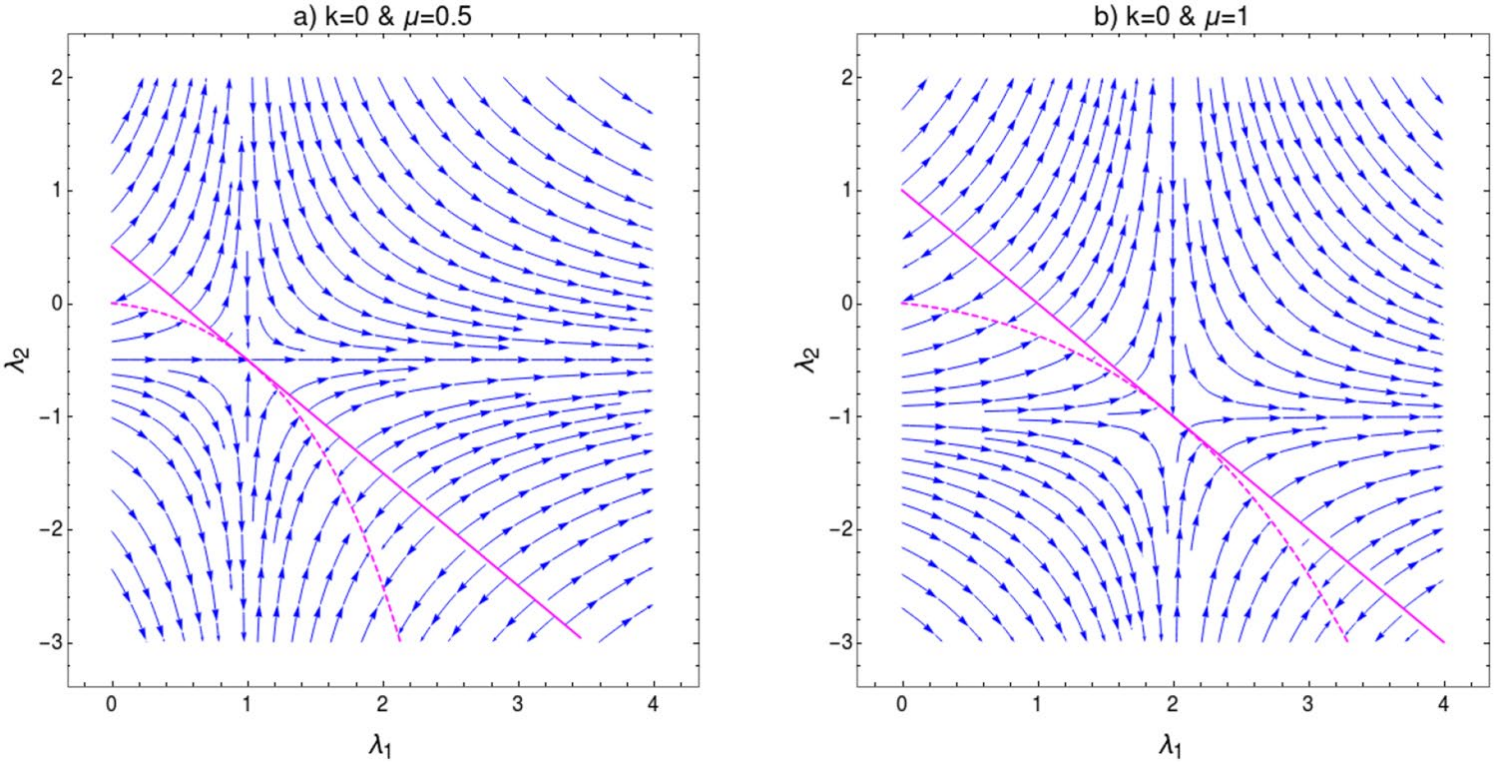
Flow diagram for $k_0=0$ in $\lambda _1$-$\lambda _2$ plane for (**a**) $\mu =0.5$ and (**b**) $\mu =1$. The RG flow directions are pointed by the arrows. The critical lines are shown as solid lines and fixed lines as dashed lines. Analyzing RG flow, distinct topological phases and the transition between them can be understood.

One can observe critical line and fixed line respectively at $\lambda _2=\mu -\lambda _1$ and $\lambda _2=\frac{\lambda _1(\lambda _1+\mu )}{\lambda _1-8\mu }$. RG flow lines for the coupling parameters $\lambda _1$ and $\lambda _2$ are depicted in Fig. 6 for $k_0=0$. It consists of two figures for different values of $\mu$. In each figure the quantum critical line and fixed line are represented as solid and dashed lines respectively. Direction of the RG flow, in the $\lambda _1$-$\lambda _2$ plane, is shown by the arrows, which signals the presence of critical and fixed lines. The critical line is denoted by solid line in the flow diagram which traces a line $\lambda _2=\mu -\lambda _1$ as predicted analytically. This line distinguish between, $w=0$ and $w=1$ gapped phases for $\lambda _1<2\mu$ and $w=2$ and $w=1$ gapped phases for $\lambda _1>2\mu$ for $\mu \ne 0$. The RG flow of coupling parameters $\lambda _1$ and $\lambda _2$ flows away from the critical line and towards the stable fixed line as shown in Fig. 6a,b. One can dubiously distinguish between $w=0$ and $w=2$ gapped phases based on the flow lines, which flows towards $\lambda _1=2\mu$ in $w=2$ phase and towards the fixed line in $w=0$ phase.

Multi-critical point appear exactly at the intersection of critical and fixed lines, i.e at the point $(\lambda _1,\lambda _2)=(2\mu ,-\mu )$. This intersection point can be obtained analytically by equating critical and fixed line equations, which yield a quadratic equation $\lambda _1^2-4\mu \lambda _1+4\mu ^2=0$. The solution of this quadratic equation is $\lambda _1=2\mu$ which is the multi-critical point for the HSP $k_0=0$. The curvature function is found to be diverging at this point. This multi-critical point distinguish the critical phases $\lambda _1<2\mu$ and $\lambda _1>2\mu$ on the critical line, whose physics can also be captured by the CRG method which is discussed in the next section.

In order to show the distinct nature of CP-1 and CP-2, we calculate the critical exponents, explained in Eq. (13), and characterize their universality classes. Set of critical exponents $(z,\nu ,\gamma )$ characterize the critical phases which governs the transition between $w=0$ and $w=1$ as well as $w=2$ and $w=1$ gapped phases. To calculate these critical exponents we first expand the Hamiltonian terms $\chi _z$ and $\chi _y$ from Eq. (3), around the HSP $k_0=0$.

21 $$\begin{aligned} \chi _z&= (2\mu -2\lambda _1-2\lambda _2)+\frac{(8\lambda _2+2\lambda _1)}{2} \delta k^2 \end{aligned}$$

22 $$\begin{aligned} \chi _y&= (4\lambda _2+2\lambda _1)\delta k, \end{aligned}$$

where $(2\mu -2\lambda _1-2\lambda _2)=\delta g$, such that $F(k_0,\delta g) = F_0|\delta g|^{-\gamma }$ and $\xi = \xi _0 |\delta g|^{-\nu }$. We substitute $B=\frac{(8\lambda _2+2\lambda _1)}{2}$ and $A=(4\lambda _2+2\lambda _1)$ and write the Berry connection in Ornstein-Zernike form in Eq. (9) as (refer to “Method” section for details)

23 $$\begin{aligned} F(k,\delta g)= \frac{\left( \frac{2BA\delta k^2-A(\delta g+B\delta k^2)}{\delta g^2}\right) }{1 + \frac{(2 \delta g B+A^2)}{\delta g^2} \delta k^2 + \frac{B^2}{\delta g^2}\delta k^4 } = \frac{F(k_0,\delta g)}{1+\xi ^2 \delta k^2+\xi ^4\delta k^4}. \end{aligned}$$

here we observe that among the coefficients of $\delta k^2$, the second term diverges more quickly and becomes dominant as we approach QCP. For transition between gapped phases $w=0$ and $w=1$, coefficient $\delta k^2$ term dominates over the coefficient $\delta k^4$ term implying $\xi \propto |\delta g|^{-1}$, thus the correlation length and dynamical critical exponents $\nu =1$ and $z=1$ respectively. For transition between gapped phases $w=2$ and $w=1$, coefficient $\delta k^4$ term dominates over the coefficient $\delta k^2$ term implying $\xi \propto |\delta g|^{-\frac{1}{2}}$, thus the critical exponents can be obtained as $\nu =\frac{1}{2}$ and $z=2$. The curvature function at the HSP $k_0=0$ can be obtained as $F(k_0,\delta g)= \frac{2(\lambda _1+2\lambda _2)}{\delta g}$. As we approach critical line $\lambda _2=\mu -\lambda _1$ the curvature function $F(k_0,\delta g) \propto |\delta g|^{-1}$ implying the curvature function critical exponent to be $\gamma =1$.

Summarizing above results suggest that the set of critical exponents for CP-1 between $w=0 \quad \text {and} \quad w=1$ are $(\nu ,z,\gamma )=(1,1,1)$ and for CP-2 between $w=2 \quad \text {and} \quad w=1$ are $(\nu ,z,\gamma )=(\frac{1}{2},2,2)$. This clearly indicate that the two gapless phases belong to different universality classes. There is a TQPT between these two gapless phases through multi-critical point which we discuss in the next section. This result coincide with the results that we obtained from energy dispersion analysis.

Note that for CP-1 the scaling law in Eq. (15) is obeyed, while for CP-2 it is violated. The dynamical critical exponent is found to take the value $z=1$ for CP-1 since the spectra is linear in *k* around the gap closing point. In the case of CP-2, the spectra is found to be quadratic in *k* around the gap closing point which yields $z=2$. For this case one can write an effective form of Eq. (23) around the HSP as

24 $$\begin{aligned} F(k,\delta g)=\frac{F(k_0,\delta g)}{(1+\xi ^4\delta k^4)}. \end{aligned}$$

Integrating this over its width $\xi _i^{-1}$ for the conservation of topological invariant, yields the scaling law $\gamma =2\sum \nolimits _{i=1}^{D} \nu _i$. Thus when $z=2$ the scaling law will get modified into $\gamma =2\nu$ for 1D systems (refer to “Method” section for details).

**Figure 7:**
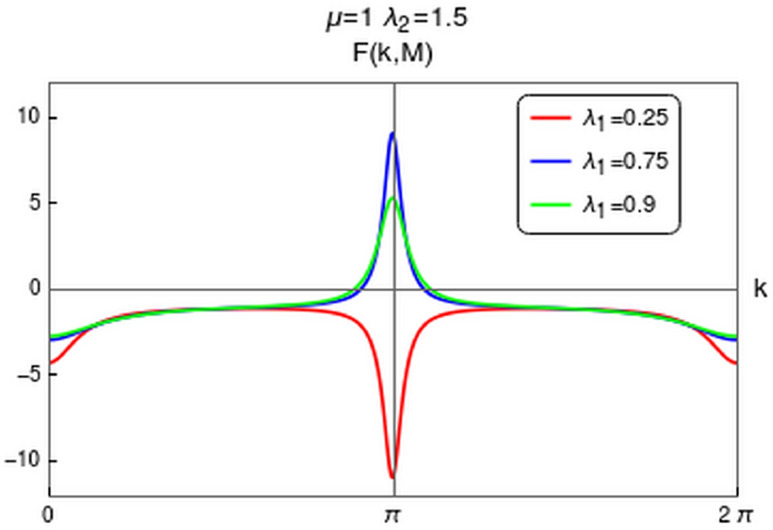
The behavior of the curvature function around the HSP $k_0=\pi$ for $\lambda _2>0$. Several values of $\lambda _1$, around the critical value $\lambda _1=0.5$, are plotted at $\mu =1$ and $\lambda _2=1.5$. Curvature function shows suitable behavior to perform CRG as it diverges at HSP on approaching critical point.

In order to verity this modification in scaling law, we perform the CRG for the HSP $k=\pi$ which address the topological transition between gapped phases $w=2$ and $w=1$ for $\lambda _2>0$. This transition happens through the critical line $\lambda _2=\mu +\lambda _1$. As we approach this QCP the curvature function in Eq. (16), diverges at the HSP $k_0=\pi$ as shown in Fig. 7 and takes the Ornstein-Zernike form around this HSP. RG flow equations for the coupling parameters $\lambda _1$, $\lambda _2$ and $\mu$ can be derived as (refer to “Method” section for a detailed derivation)

25 $$\begin{aligned} \frac{d\lambda _1}{dl}= & {} \frac{{\lambda _1 }^2+{\lambda _1} ({\lambda _2 }-\mu )+8 {\lambda _2 } \mu }{2 ({\lambda _1 }-{\lambda _2 }+\mu )}, \end{aligned}$$

26 $$\begin{aligned} \frac{d\lambda _2}{dl}= & {} -\frac{({\lambda _2 }+\mu ) \left( {\lambda _1 }^2+{\lambda _1 } ({\lambda _2 }-\mu )+8 {\lambda _2 } \mu \right) }{2 ({\lambda _1 }+2 \mu ) ({\lambda _1 }-{\lambda _2 }+\mu )}, \end{aligned}$$

27 $$\begin{aligned} \frac{d\mu }{dl}= & {} -\frac{({\lambda _2 }+\mu ) \left( {\lambda _1 }^2+{\lambda _1 } ({\lambda _2 }-\mu )+8 {\lambda _2 } \mu \right) }{2 ({\lambda _1 }-2 {\lambda _2 }) ({\lambda _1 }-{\lambda _2 }+\mu )}. \end{aligned}$$

For a constant value of $\mu$, Eqs. (25) and (26) satisfy the conditions

28 $$\begin{aligned} \left| \dfrac{d\lambda _1}{dl}\right| =\left| \dfrac{d\lambda _2}{dl}\right| = \infty \quad \text {and} \quad \left| \dfrac{d\lambda _1}{dl}\right| =\left| \dfrac{d\lambda _2}{dl}\right| = 0. \end{aligned}$$

**Figure 8:**
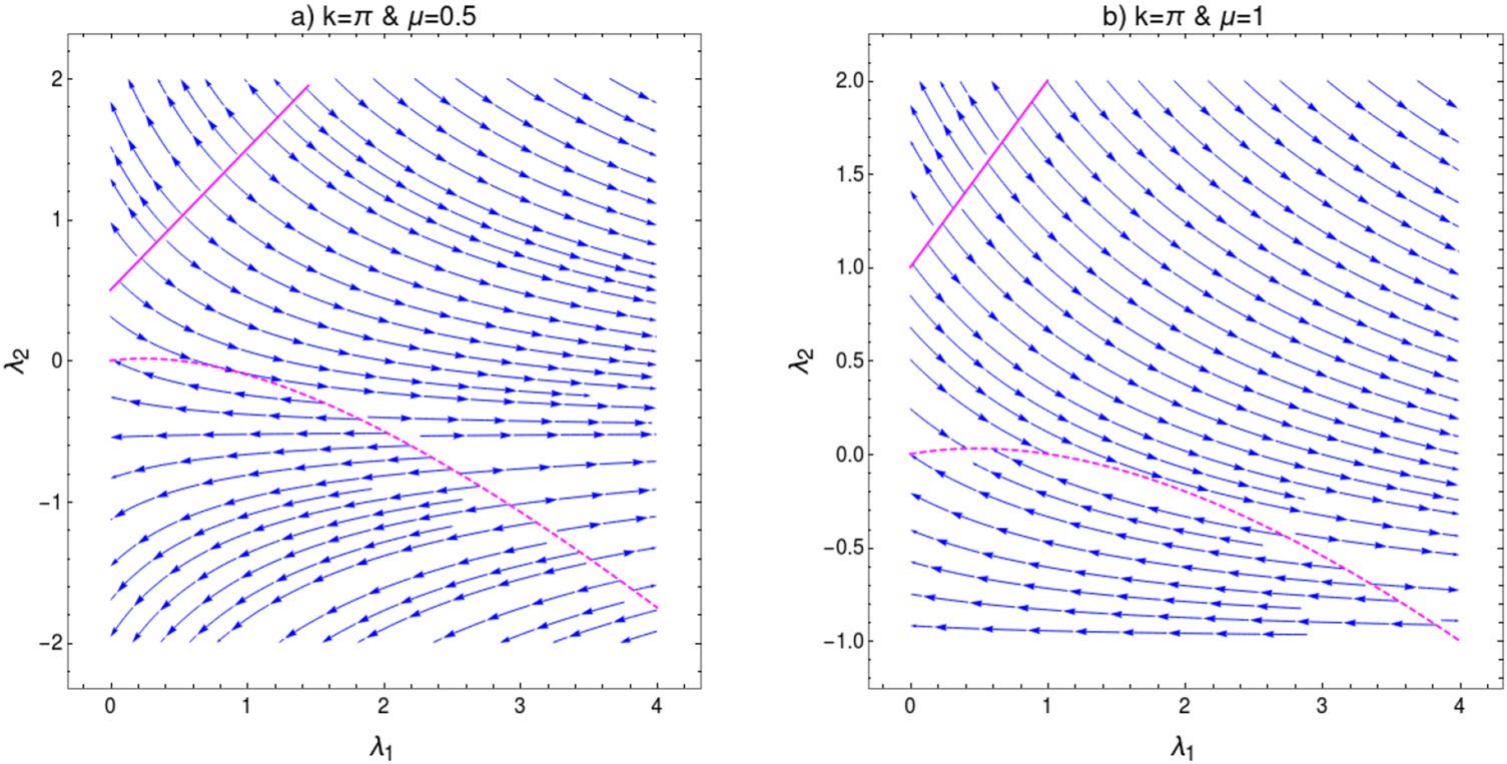
Flow diagram for $k=\pi$ in $\lambda _1$-$\lambda _2$ plane for (**a**) $\mu =0.5$ and (**b**) $\mu =1$. The RG flow directions are pointed by the arrows. The critical and fixed lines are shown as solid and dashed lines respectively.

The critical line and fixed line can be found at $\lambda _2=\mu +\lambda _1$ and $\lambda _2=\frac{\lambda _1(\mu -\lambda _1)}{8\mu +\lambda _1}$ respectively. The RG flow diagram for coupling parameters at $k_0=\pi$ is shown in Fig. 8. It consists of two figures for different values of $\mu$. In each figure the quantum critical line and fixed line are represented as solid and dashed lines respectively. The critical line $\lambda _2=\mu +\lambda _1$, represented as solid line in the flow diagram, distinguish between $w=2$ and $w=1$ gapped phases. The RG flow lines flowing away from this critical line indicate the TQPT between these gapped phases. The fixed lines are represented as dashed curve in Fig. 8a,b. A part of this fixed line is stable where flow lines flows towards it and a part is unstable where flows are away from it for $\mu \ne 0$. The intersection of these critical and fixed lines can be obtained analytically by equating critical and fixed line equations. This yield $\lambda _1=-\mu$, which indicate there is no intersection point for positive $\mu$ or $\lambda _1$ values.

We verify the value of critical exponent $\nu$ using Berry connection approach. Expanding the Hamiltonian terms $\chi _z$ and $\chi _y$ of Eq. (3) around the HSP $k_0=\pi$ upto first order in *k* and writing the Berry connection $F(k_0,\mathbf {M})$ in the form of Eq. (9) yields (refer to “Method” section for details)

29 $$\begin{aligned} F(k,\delta g)= \frac{\left( \frac{A}{\delta g}\right) }{1 + \frac{(A^2)}{\delta g^2} \delta k^2} = \frac{F(k_0,\delta g)}{1+\xi ^2 \delta k^2}, \end{aligned}$$

where $A=(4\lambda _2-2\lambda _1)$ and $\delta g=(2\mu +2\lambda _1-2\lambda _2)$. This clearly indicate $\xi \propto |\delta g|^{-1}$, which implies the correlation length critical exponent $\nu =1$. The curvature function at the HSP $k_0=\pi$ can be written as $F(k_0,\delta g)= \frac{2(2\lambda _2-\lambda _1)}{\delta g}$. As we approach the critical line $\lambda _2=\mu +\lambda _1$, curvature function is $F(k_0,\delta g)\propto |\delta g|^{-1}$ which implies the value of $\gamma =1$. Thus we obtain a set of critical exponents i.e, $(z,\nu ,\gamma )=(1,1,1)$ for the transition between gapped phases at $k_0=\pi$. Note that the critical exponents obey the scaling law in Eq. (15). Since the spectra on the critical line is linear around the gap closing point with $z=1$, the scaling law $\nu =\gamma$ is obeyed. Even though there is a transition between $w=1$ and $w=2$ gapped phases for both $k_0=0$ and $k_0=\pi$ HSPs, the nature of energy spectra, critical theory and the scaling of curvature function are different. This results in the modified scaling law observed previously for CP-2 at $k_0=0$.

#### CRG for the transition between gapless phases

In this section we discuss the topological transition between the gapless phases through multi-critical point on the critical line $\lambda _2=\mu -\lambda _1$. The gapless phases CP-1 and CP-2 are found to have different set of critical exponents. The nature of transition between these two distinct gapless phases is indeed topological and occurs through the multi-critical point ‘b’ (see Fig. 1). We perform CRG again and derive RG equations and critical exponents to prove the existence of topological transition between gapless phases and also to characterize the critical behavior at the multi-critical point.

**Figure 9:**
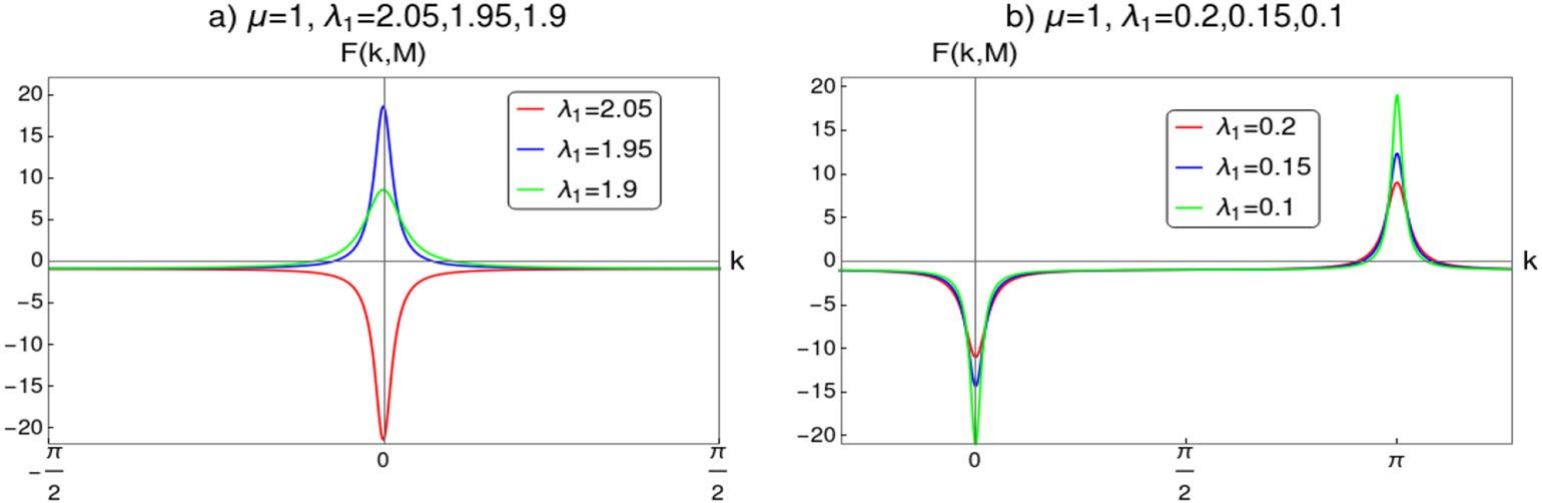
Curvature function $F(k,\mathbf {M})$ near the multi-critical points. (**a**) Curvature function is plotted around the multi-critical point ‘b’ in the phase diagram, which distinguish between the distinct gapless phases (CP-1 and CP-2) on the critical line $\lambda _2=\mu -\lambda _1$. (**b**) Curvature function is plotted around the multi-critical point ‘a’ in the phase diagram. Both are plotted for several values of $\lambda _1$ at $\mu =1$.

Curvature function on the critical line $\lambda _2=\mu -\lambda _1$ can be obtained as

30 $$\begin{aligned} \begin{aligned} F(k,\mathbf {M})&= \dfrac{d\phi _k}{dk}\\&= \dfrac{d}{dk}\left[ \tan ^{-1}\left( \frac{2 (\mu -\lambda _1) \sin (2 k) +2 \lambda _1 \sin (k)}{2 \mu -2 (\mu -\lambda _1) \cos (2 k) -2 \lambda _1 \cos (k)}\right) \right] \\&= -\frac{\lambda _1 (\lambda _1-2 \mu )}{2 \left( \lambda _1^2-2 \lambda _1 \mu +2 \mu ^2 + 2 \mu (\mu -\lambda _1) \cos (k) \right) }-1, \end{aligned} \end{aligned}$$

where $\mathbf {M}= \left\{ \mu , \lambda _1 \right\}$. Fig. 9a shows $F(k,\mathbf {M})$ for the transition between gapless phases through multi-critical point. Surprisingly the curvature function tend to diverge as we approach the multi-critical point. For the parameter value $\mu =1$ multi-critical point is obtained at the critical value $\lambda _1=2$. Curvature function shows diverging peak as we approach critical value and flips sign across it. This behavior of the curvature function allow one to perform CRG to understand the topological transition between gapless phases.

The behavior of curvature function at the multi-critical point ‘a’ is shown in Fig. 9b. It is a trivial multi-critical point at which two critical line, $\lambda _2=\mu +\lambda _1$ and $\lambda _2=\mu -\lambda _1$ meet. Hence, as we approach this multi-critical point from either directions the curvature function diverges at both HSPs $k_0=0$ and $k_0=\pi$. This multi-critical point preserve Lorentz invariance and no topological transition occurs between gapless phases as in the case of the multi-critical point ‘b’.

The RG flow equations, which signals the topological transition between the gapless phases through multi-critical point, for the coupling parameters $\lambda _1$ and $\mu$, can be derived as (refer to “Method” section for a detailed derivation)

31 $$\begin{aligned} \frac{d\lambda _1}{dl}=-\frac{\lambda _1(\lambda _1-\mu )}{2(\lambda _1-2\mu )} \quad \text {and} \quad \frac{d\mu }{dl}=-\frac{\mu (\mu -\lambda _1)}{2(\lambda _1-2\mu )}. \end{aligned}$$

One can immediately spot a critical line for $\lambda _1=2\mu$ and a fixed line for $\lambda _1=\mu$ at which the RG equations satisfy the condition

32 $$\begin{aligned} \left| \dfrac{d\lambda _1}{dl}\right| =\left| \dfrac{d\mu }{dl}\right| \rightarrow \infty \quad \text {and} \quad \left| \dfrac{d\lambda _1}{dl}\right| =\left| \dfrac{d\mu }{dl}\right| \rightarrow 0. \end{aligned}$$

**Figure 10:**
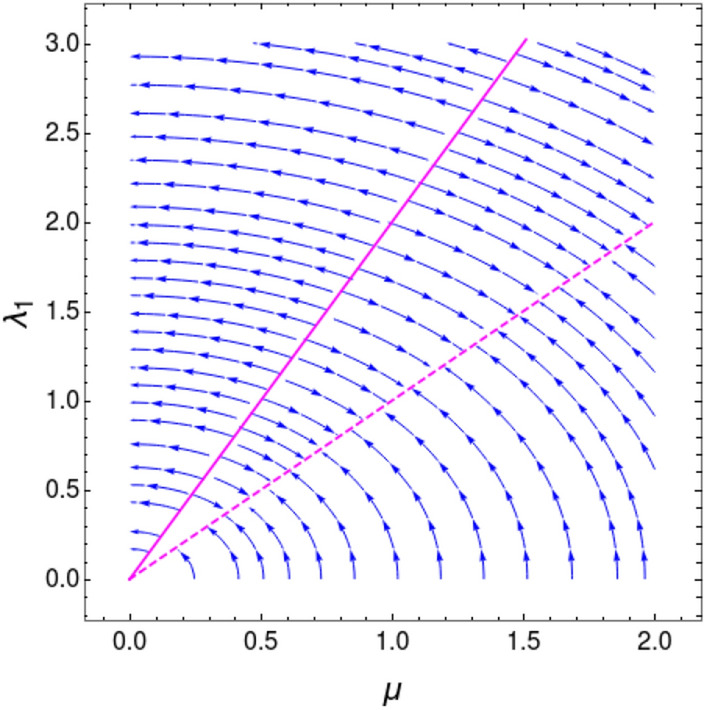
RG flow lines on the critical line $\lambda _2=\mu -\lambda _1$. RG flow are away from the line $\lambda _1=2\mu$ (solid line) and towards the line $\lambda _1=\mu$ (dashed line) which are critical and fixed lines respectively.

The RG flow lines for the coupling parameters $\lambda _1$ and $\mu$ is shown in Fig. 10. Quantum critical line and fixed line are represented as solid and dashed lines respectively. The line $\lambda _1=2\mu$, solid line in Fig. 10, indicate the multi-critical points for different values of $\mu$. This line distinguish between the $w=0$ (CP-1) and $w=1$ (CP-2) gapless phases on the critical line $\lambda _2=\mu -\lambda _1$. Therefore it indicate the TQPT between these gapless phases through the multi-critical point. The dashed line in Fig. 10, $\lambda _1=\mu$ represent fixed points in the flow diagram. The intersection of critical and fixed lines can be obtained analytically at $\mu =0$ and also can be observed at the same point in the flow diagram.

To characterize the critical behavior at the multi-critical point we calculate the critical exponents $(z,\nu ,\gamma )$ as done in the case of gapped phases. Critical exponents can be calculated by expanding the Hamiltonian terms $\chi _z$ and $\chi _y$ from Eq. (3) on the critical line $\lambda _2=\mu -\lambda _1$, around the HSP $k_0=0$ upto third order.

33 $$\begin{aligned} \chi _z&= \left( \frac{8\mu -6\lambda _1}{2}\right) \delta k^2 = B \delta k^2, \end{aligned}$$

where $$B= \left( \frac{8\mu -6\lambda _1}{2}\right) $$, and

34 $$\begin{aligned} \chi _y&=-2(\lambda _1-2\mu )\delta k - \left( \frac{16\mu + 18\lambda _1}{6}\right) \delta k^3 = -2\delta g \delta k- A \delta k^3, \end{aligned}$$

where $(\lambda _1-2\mu )=\delta g$ and $A=\left( \frac{16\mu + 18\lambda _1}{6}\right)$. Now the Berry connection can be written as (refer to “Method” section for details)

35 $$\begin{aligned} F(k,\delta g)= \frac{\left( \frac{-2B\delta g \delta k^2 + B A \delta k^4}{4 \delta g^2 \delta k^2}\right) }{1+\left( \frac{A^2+4\delta g B}{4 \delta g^2}\right) \delta k^2 + \left( \frac{B^2}{4 \delta g^2} \right) \delta k^4 }=\frac{F(k_0,\delta g)}{1+\xi ^2\delta k^2+\xi ^4 \delta k^4}. \end{aligned}$$

For different parameter values on the critical line, we observe the coefficient of $\delta k^4$ is dominant over $\delta k^2$. This implies the correlation length $\xi \propto |\delta g|^{-\frac{1}{2}}$, suggesting the correlation length exponent and dynamical critical exponents to be $\nu =\frac{1}{2}$ and $z=2$ respectively. To calculate the critical exponent $\gamma$ we obtain the curvature function at HSP, which has a form $F(k_0,\delta g) =\frac{4\mu -3\lambda _1}{2} |\delta g|^{-1}$. Therefore as we approach multi-critical point the curvature function critical exponent takes the value $\gamma =1$. Note that the scaling law is violated here also as in the case of the transition between the gapped phases $w=2$ and $w=1$ for $\lambda _2<0$. As proposed earlier the scaling law get modified as $\gamma =2\nu$ since the dynamical critical exponent $z=2$. Thus the critical phase at the multi-critical point, which governs the topological transition between two gapless phases on the critical line $\lambda _2=\mu -\lambda _1$, has critical exponents $(\nu ,z,\gamma )=(\frac{1}{2},2,1)$.

#### General discussions on RG flow behavior

Here we discuss the general features of RG flow of coupling parameters for gapped phases. Behavior of RG flow lines are different for different quantum critical lines i.e, for $k_0=0$ and $k_0=\pi$, shown in Figs. 6 and 8. This difference is due to the distinct nature of fixed lines for both HSPs. In Fig. 6 we observe the fixed line at $\lambda _2=\frac{\lambda _1(\lambda _1+\mu )}{\lambda _1-8\mu }$. This fixed line is stable for finite range of parameter values and flow lines flows towards it. However, it is not the same case in Fig. 8. The fixed line occurs at $\lambda _2=\frac{\lambda _1(\mu -\lambda _1)}{8\mu +\lambda _1}$, which has both stable and unstable parts. This causes a major distortion in the RG flow on $\lambda _1$-$\lambda _2$ plane. Thus the nature of RG flow are different for different critical lines.

**Figure 11:**
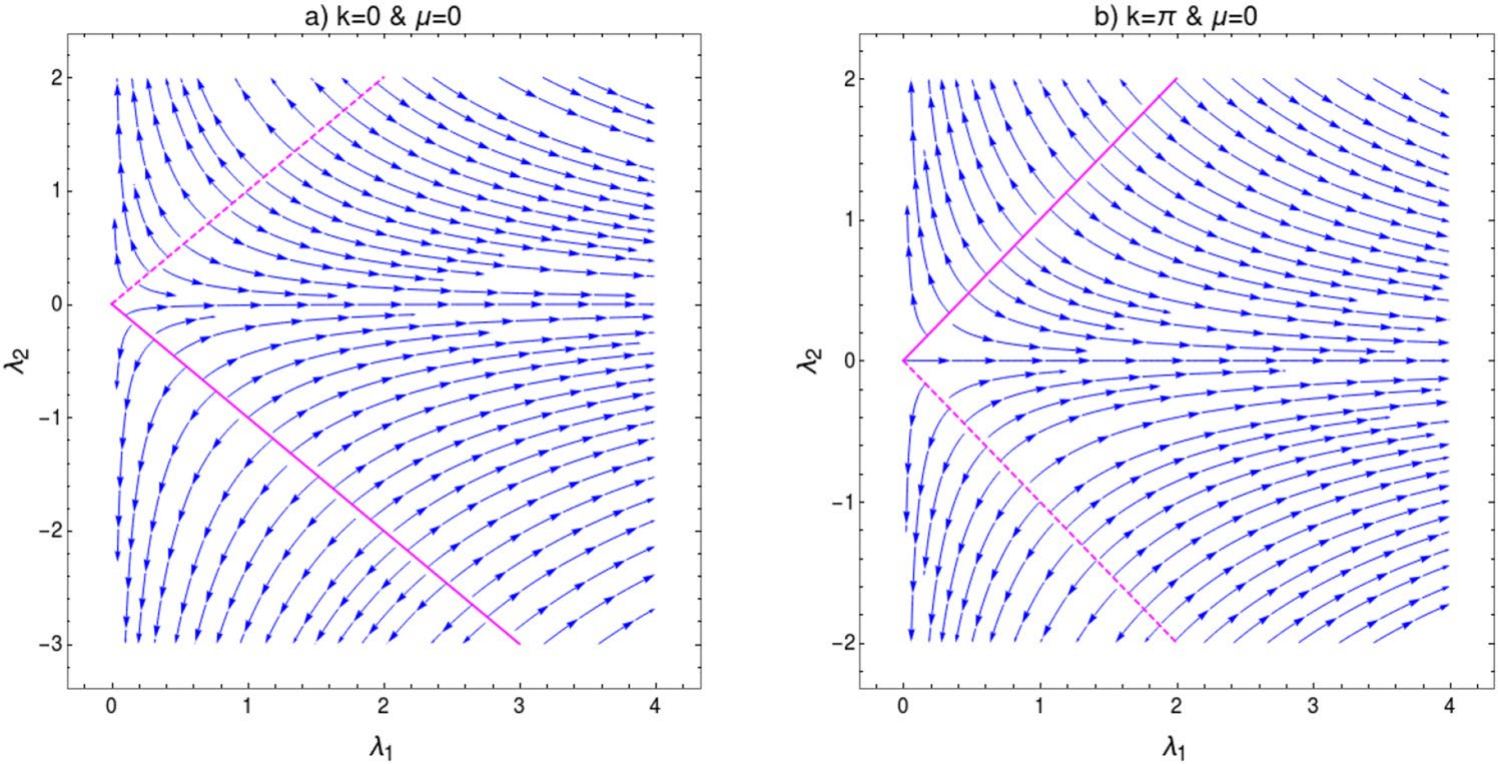
(**a**) Flow diagram for $k_0=0$ [Eqs. (17), (18)] at $\mu =0$, (**b**) Flow diagram for $k_0=\pi$ [Eqs. (25), (26)] at $\mu =0$. The RG flow directions are pointed by the arrows. The critical lines are shown as solid lines and fixed lines as dashed lines.

An interesting point can be observed when one set the parameter $\mu =0$. RG flow in this case is shown in Fig. 11 for both HSPs. Setting $\mu =0$, removes non-topological phase ($w=0$) completely and only topological gapped phases remain. It also eliminate the non-trivial multi-critical point along with distinct gapless phases. Hence, the RG flow at $\mu =0$ for both HSPs are similar in nature. The fixed lines for both HSPs are unstable with RG flow lines flowing away. It is interesting to note that for $k_0=0$ (Fig. 11a), the fixed line coincide with critical line for $k_0=\pi$. Similarly for $k_0=\pi$ (Fig. 11b) the fixed line coincide with the critical line for $k_0=0$.

RG flow lines in Fig. 6 shows asymptotic nature around the the line $\lambda _1=2\mu$. The flow direction is reversed on the opposite sides of the multi-critical point, which occurs at the intersection of fixed and critical lines. This nature of RG flow lines are due to the term $\lambda _1-2\mu$ in the denominator of RG equation for $\lambda _2$ in Eq. (18). This RG equation blows up for $\lambda _1=2\mu$ which accounts for the asymptotic nature of RG flow lines in Fig. 6. For $\lambda _2$ value above the multi-critical point, RG flow asymptotically increase for $\lambda _1<2\mu$ and asymptotically decrease for $\lambda _1>2\mu$. This flow directions reverses for $\lambda _2$ value below the multi-critical point. Similar nature can be expected for HSP $k_0=\pi$ around the line $\lambda _1=-2\mu$.

**Figure 12:**
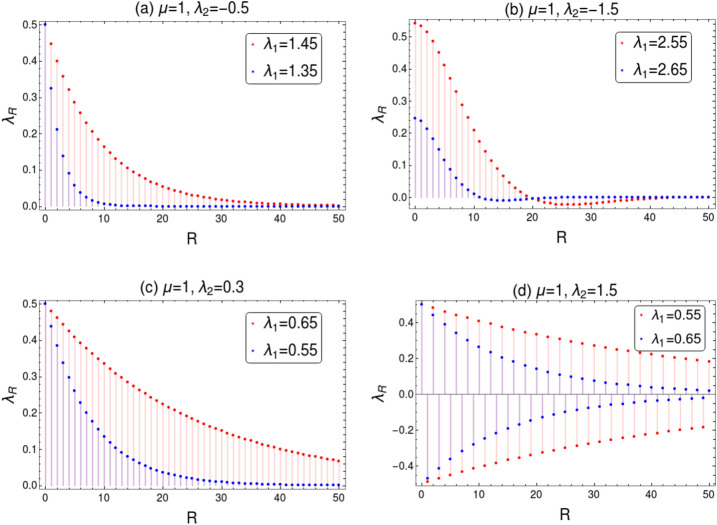
Behavior of correlation function $\lambda _R$ near quantum critical lines. (**a**) $\lambda _R$ is plotted near the critical line $\lambda _2=\mu -\lambda _1$ for the transition between $w=0$ and $w=1$ (i.e CP-1) with $\lambda _2<0$, where critical value of $\lambda _1=1.5$. (**b**) $\lambda _R$ is plotted near the critical line $\lambda _2=\mu -\lambda _1$ for the transition between $w=2$ and $w=1$ (i.e CP-2), where critical value of $\lambda _1=2.5$. (**c**) $\lambda _R$ is plotted near the critical line $\lambda _2=\mu -\lambda _1$ for the transition between $w=0$ and $w=1$ (i.e CP-1) with $\lambda _2>0$, where critical value of $\lambda _1=0.7$. (**d**) $\lambda _R$ is plotted near the critical line $\lambda _2=\mu +\lambda _1$ for the transition between $w=2$ and $w=1$, where the critical value of $\lambda _1=0.5$.

**Figure 13:**
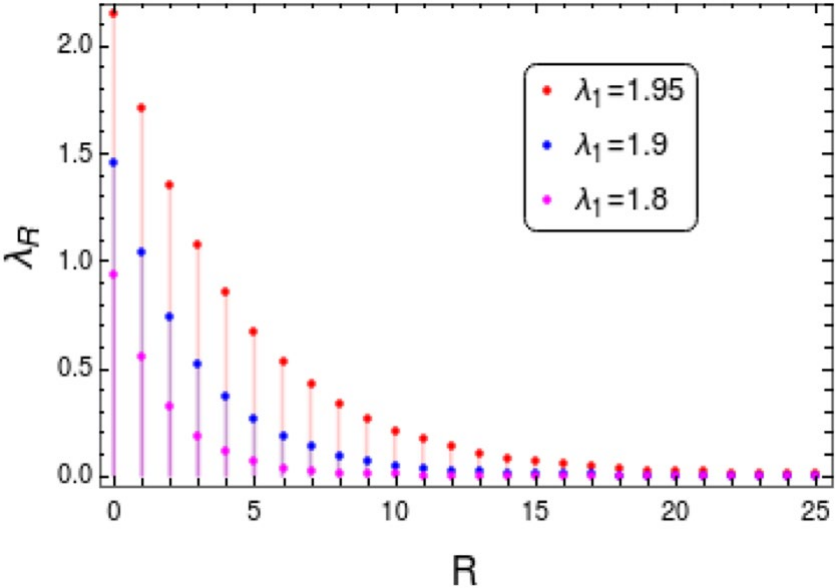
Behavior of correlation function $\lambda _R$ near the multi-critical point $\lambda _1=2\mu$. The critical value $\lambda _1=2$ with $\mu =1$.

#### Correlation function for gapped and gapless phases

Now we discuss the physical significance of correlation length as a length scale to determine the correlation between Wannier states. In the case of one dimensional systems, the curvature function is given by the Berry connection $F (k, \mathbf {M}) = \sum _{n} \left\langle u_{kn} | i \delta _k | u_{kn} \right\rangle$, where *n* is the index of all occupied bands. The Fourier transform of which gives the charge polarization correlation function ($\lambda _R$), between Wannier states at a distance *R* apart^20,27^.

36 $$\begin{aligned} \lambda _R = \int \frac{dk}{2 \pi } e^{i k.R} F (k, \mathbf {M}) = \int \frac{dk}{2 \pi } e^{i k.R} \sum _{n} \left\langle u_{kn} | i \delta _k | u_{kn} \right\rangle = \sum _{n} \left\langle Rn | r |0 n \right\rangle . \end{aligned}$$

We have two bands in our model and only the lower band $(n=1)$ is occupied. Therefore we have $\lambda _R = \left\langle R|r|0\right\rangle$, which is a measure of overlap between Wannier states at 0 and *R*. The zeroth component $\lambda _0$ is the charge polarization, which is the topological invariant. Since Wannier state $\left\langle r|R\right\rangle = W (r - R)$ is a localized function with center at *R*, the quantity $\left\langle R|r|0\right\rangle$ is expected to decay with *R* to zero.

The correlation function $\lambda _R$ can be analytically calculated in the continuous approximation for the appropriate gauge choice of Berry connection, which takes Ornstein-Zernike form. We study the behavior of $\lambda _R$ near the critical line $\lambda _2=\mu -\lambda _1$ which occurs at the HSP $k_0=0$. Since the critical line has distinct gapless phases (CP-1 and CP-2), we study the nature of $\lambda _R$ separately near these gapless phases. As we approach the CP-1 i.e, for the transition between gapped $w=0$ to $w=1$ phase, the correlation function $\lambda _R$ can be obtained as (refer to “Method” section for details)

37 $$\begin{aligned} \lambda _R=\frac{1}{2\xi } \left( \frac{2 (\lambda _1+2 \lambda _2)}{2 \mu -2 \lambda _1-2 \lambda _2}\right) \exp \left( -\frac{|R|}{\xi }\right) , \end{aligned}$$

where $\xi =\frac{2 (\lambda _1+2 \lambda _2)}{2 \mu -2 \lambda _1-2 \lambda _2}$. Similarly as we approach the CP-2 i.e, for the transition between gapped $w=1$ to $w=2$ phase, $\lambda _R$ can be obtained as (refer to “Method” section for details)

38 $$\begin{aligned} \lambda _R=\frac{1}{2\;\xi \sqrt{2}}\left( \frac{2 (\lambda _1+2 \lambda _2)}{2 \mu -2 \lambda _1-2 \lambda _2}\right) \left\{ \sin \left( \frac{\left| R\right| }{\sqrt{2}\;\xi }\right) +\cos \left( \frac{\left| R\right| }{\sqrt{2}\;\xi }\right) \right\} \exp {\left( - \frac{|R|}{\sqrt{2} \;\xi }\right) } , \end{aligned}$$

where $\xi = \sqrt{\frac{2 \lambda _1+8 \lambda _2}{2 (2 \mu -2\lambda _1-2\lambda _2)}}$. Behavior of correlation function near the critical lines between distinct gapped phases is depicted in Fig. 12. Figure 12a shows the decay in the correlation function in Eq. 37 as we approach a critical point at $\lambda _1=1.5$ on CP-1. We observe the decay length of the $\lambda _R$ is shorter for the parameter value away from the critical value and it gets longer as we approach the critical point. In other words the correlation function decays slower near the critical line as the decay is sharp deep inside the gapped phase. Similar behavior can be observed for the transition across CP-2 as shown in Fig. 12b. In this case the critical point is at $\lambda _1=2.5$. $\lambda _R$ shows sharp decay for the parameter value away from the critical value and the decay length is longer as we approach the critical point. This indicate the TQPT between the gapped phases as this behavior of correlation function is universal around a QCP. Note that for $w= 0$ gapped phase, $\lambda _2$ range from $-1$ to 1 (see Fig. 1). In this range of $\lambda _2$, we consider one attractive (− ve) and the other one repulsive (+ ve) coupling. Figure 12a is plotted for attractive coupling of $\lambda _2$ and Fig. 12c is plotted for repulsive coupling of $\lambda _2$. The critical value of $\lambda _1=0.7$ near to which $\lambda _R$ decay slowly and sharp decay can be observed for the value away from critical value. We observe the decay in $\lambda _R$ is much slower in the repulsive case than in the attractive case at the same distance from the critical line.

The topological transition across the critical line $\lambda _2=\mu +\lambda _1$ can also be observed in terms $\lambda _R$. This critical line corresponds to the transition between gapped phases with $w=2$ and $w=1$. Behavior of $\lambda _R$ for the HSP $k_0=\pi$ can be obtained as (refer to “Method” section for details)

39 $$\begin{aligned} \lambda _R=\frac{(-1)^R}{2\;\xi }\left( \frac{2 (2 \lambda _2 -\lambda _1)}{2 \lambda _1-2 \lambda _2 +2 \mu }\right) \exp \left( -\frac{|R|}{\xi }\right) , \end{aligned}$$

where $\xi =\left( \frac{4 \lambda _2-2 \lambda _1}{2 \mu +2 \lambda _1 -2 \lambda _2 }\right)$. Figure 12d shows oscillatory behavior of $\lambda _R$ close to the critical point at $\lambda _1=0.5$ on the critical line $\lambda _2=\mu +\lambda _1$. We observe that the amplitude of the oscillation decreases, which indicate the decay in $\lambda _R$. This decay gets slower as we approach the critical point as in Fig. 12d. This clearly confirms the presence of TQPT across the critical point between the gapped phases $w=1$ and $w=2$.

Behavior of correlation function $\lambda _R$ near a critical point signals the TQPT successfully. Therefore we analyze the same universal property of $\lambda _R$ for the transition between gapless phases CP-1 and CP-2. The analytical expression for the gapless excitation of the correlation function $\lambda _R$ can be obtained as (refer to “Method” section for details)

40 $$\begin{aligned} \lambda _R=\frac{1}{2\;\xi \sqrt{2}}\left( \frac{4 \mu -3 \lambda _1}{2 (\lambda _1-2 \mu )}\right) \left\{ \sin \left( \frac{\left| R\right| }{\sqrt{2}\;\xi }\right) +\cos \left( \frac{\left| R\right| }{\sqrt{2}\;\xi }\right) \right\} \exp {\left( -\frac{|R|}{\xi }\right) }, \end{aligned}$$

where $\xi = \sqrt{\frac{8 \mu -6 \lambda _1}{4 (\lambda _1-2\mu )}}$. Figure 13 shows the behavior of $\lambda _R$ as we approach the multi-critical point at $\lambda _1=2$. $\lambda _R$ decays sharply deep within the gapless phase and the decay length increases as the $\lambda _1$ value approaches critical point. The decay tends to slow down with longer decay length for the value close to critical point. This behavior of $\lambda _R$ near the multi-critical point is similar to the cases of gapped phases. One can conclude from the behavior of $\lambda _R$ in Fig. 13 that it clearly indicate the presence of TQPT across the multi-critical point between the gapless phases CP-1 and CP-2.

## Discussion

The theory of critical phenomena and curvature function renormalization scheme, developed for the topological phase transitions, provides an alternative platform to understand the transition between gapped phases against the conventional theory on topological invariant. We have shown explicitly that these tools can also be extended for the characterization of topological quantum phase transition occurring between gapless phases. The two distinct gapless phases of our model Hamiltonian has been analyzed and they were found to belong to different universality classes based on the values of critical exponents. Among the three quantum critical lines of the model Hamiltonian, two are topological in nature and also capture the essential TQPT across the gapless topological quantum critical line. This interesting feature is absent in the original Kitaev chain. CRG analysis confirmed the presence of topological quantum phase transition between the gapless phases through the non-trivial multi-critical point. We have shown explicitly the break down of Lorentz invariance at the topological multi-critical point. The values of critical exponents revealed that the transition is in the Lifshitz universality class. We have performed the calculation of Wannier state correlation function for the TQPT between gapped and gapless phases. Decrease in the decay rate of correlation function as we approach multi-critical point revealed the presence of TQPT between gapless phases.

## Methods

### Derivation of CRG equations

#### For gapped phases

Here, we derive the RG equations for $k_0=0$. Referring the generic form of the RG equation in Eq. (12) we obtain three RG equations corresponding to the parameters. Curvature function can be obtained as

41 $$\begin{aligned} F(k,\mathbf {M})= \frac{\lambda _1 \cos (k) (\mu -3 \lambda _2)+2 \lambda _2 \mu \cos (2 k)-\lambda _1^2-2 \lambda _2^2}{2 \lambda _1 \cos (k) (\lambda _2-\mu )-2 \lambda _2 \mu \cos (2 k)+\lambda _1^2+\lambda _2^2+\mu ^2}, \end{aligned}$$

where $\mathbf {M}= \left\{ \lambda _1,\lambda _2,\mu \right\}$. Second derivative of $F(k,\mathbf {M})$ at $k_0=0$ is

42 $$\begin{aligned} \partial _k^2 F(k,\mathbf {M})|_{k=0}=\frac{({\lambda _2}+\mu ) \left( {\lambda _1}^2+{\lambda _1} (\mu -{\lambda _2})+8 {\lambda _2 } \mu \right) }{({\lambda _1 }+{\lambda _2 }-\mu )^3}. \end{aligned}$$

Derivative of the curvature function at $k_0=0$ with respect to the parameters $\lambda _1,\lambda _2$ and $\mu$ are correspondingly

43 $$\begin{aligned} \partial _{\lambda _1}F(0,\mathbf {M})&= \frac{{\lambda _2 }+\mu }{({\lambda _1 }+{\lambda _2 }-\mu )^2}, \end{aligned}$$

44 $$\begin{aligned} \partial _{\lambda _2}F(0,\mathbf {M})&= \frac{2 \mu -{\lambda _1 }}{({\lambda _1 }+{\lambda _2 }-\mu )^2}, \end{aligned}$$

45 $$\begin{aligned} \partial _{\mu }F(0,\mathbf {M})&= -\frac{{\lambda _1 }+2 {\lambda _2 }}{({\lambda _1 }+{\lambda _2 }-\mu )^2}. \end{aligned}$$

This gives three RG equations for the parameters as

46 $$\begin{aligned} \frac{d\lambda _1}{dl}&= \frac{1}{2} \frac{({\lambda _2 }+\mu ) \left( {\lambda _1 }^2+{\lambda _1 } (\mu -{\lambda _1 })+8 {\lambda _1 } \mu \right) ({\lambda _1 }+{\lambda _2 }-\mu )^2}{({\lambda _1 }+{\lambda _2 }-\mu )^3 ({\lambda _2 }+\mu )}\nonumber \\&=\frac{{\lambda _1 }^2+{\lambda _1} (\mu -{\lambda _2 })+8 {\lambda _2 } \mu }{2 ({\lambda _1 }+{\lambda _2 }-\mu )}, \end{aligned}$$

47 $$\begin{aligned} \frac{d\lambda _2}{dl}&=\frac{1}{2}\frac{({\lambda _2}+\mu ) \left( {\lambda _1 }^2+{\lambda _1 } (\mu -{\lambda _2 })+8 {\lambda _2 } \mu \right) ({\lambda _1 }+{\lambda _2 }-\mu )^2}{({\lambda _1 }+{\lambda _2 }-\mu )^3 (2 \mu -{\lambda _1 })}\nonumber \\&=-\frac{({\lambda _2 }+\mu ) \left( {\lambda _1 }^2+{\lambda _1 } (\mu -{\lambda _2 })+8 {\lambda _2 } \mu \right) }{2 ({\lambda _1 }-2 \mu ) ({\lambda _1 }+{\lambda _2 }-\mu )}, \end{aligned}$$

48 $$\begin{aligned} \frac{d\mu }{dl}&=-\frac{1}{2} \frac{({\lambda _2 }+\mu ) \left( {\lambda _1 }^2+{\lambda _1 } (\mu -{\lambda _2 })+8 {\lambda _2 } \mu \right) ({\lambda _1}+{\lambda _2 }-\mu )^2}{({\lambda _1 }+{\lambda _2 }-\mu )^3 ({\lambda _1 }+2 {\lambda _2 })}\nonumber \\&=-\frac{({\lambda _2 }+\mu ) \left( {\lambda _1 }^2+{\lambda _1 } (\mu -{\lambda _2 })+8 {\lambda _2 } \mu \right) }{2 ({\lambda _1 }+2 {\lambda _2 }) ({\lambda _1 }+{\lambda _2 }-\mu )}. \end{aligned}$$

Following the similarly procedure one can obtain RG equations for HSP $k_0=\pi$. Second derivative of $F(k,\mathbf {M})$ is taken at $k_0=\pi$

49 $$\begin{aligned} \partial _k^2 F(k,\mathbf {M})|_{k=\pi }=-\frac{({\lambda _2}+\mu ) \left( {\lambda _1}^2+{\lambda _1} ({\lambda _2}-\mu )+8 {\lambda _2 } \mu \right) }{({\lambda _1 }-{\lambda _2 }+\mu )^3}. \end{aligned}$$

Derivative of $F(k,\mathbf {M})$ at $k_0=\pi$ with respect to the parameters are

50 $$\begin{aligned} \partial _{\lambda _1}F(\pi ,\mathbf {M})&= -\frac{{\lambda _2 }+\mu }{({\lambda _1 }-{\lambda _2 }+\mu )^2}, \end{aligned}$$

51 $$\begin{aligned} \partial _{\lambda _2}F(\pi ,\mathbf {M})&= \frac{2 \mu +{\lambda _1 }}{({\lambda _1 }-{\lambda _2 }+\mu )^2}, \end{aligned}$$

52 $$\begin{aligned} \partial _{\mu }F(\pi ,\mathbf {M})&= \frac{{\lambda _1 }-2 {\lambda _2 }}{({\lambda _1 }-{\lambda _2 }+\mu )^2}. \end{aligned}$$

After few steps of calculation one can arrive at the RG equations

53 $$\begin{aligned} \frac{d\lambda _1}{dl}= & {} \frac{{\lambda _1 }^2+{\lambda _1} ({\lambda _2 }-\mu )+8 {\lambda _2 } \mu }{2 ({\lambda _1 }-{\lambda _2 }+\mu )}, \end{aligned}$$

54 $$\begin{aligned} \frac{d\lambda _2}{dl}= & {} -\frac{({\lambda _2 }+\mu ) \left( {\lambda _1 }^2+{\lambda _1 } ({\lambda _2 }-\mu )+8 {\lambda _2 } \mu \right) }{2 ({\lambda _1 }+2 \mu ) ({\lambda _1 }-{\lambda _2 }+\mu )}, \end{aligned}$$

55 $$\begin{aligned} \frac{d\mu }{dl}= & {} -\frac{({\lambda _2 }+\mu ) \left( {\lambda _1 }^2+{\lambda _1 } ({\lambda _2 }-\mu )+8 {\lambda _2 } \mu \right) }{2 ({\lambda _1 }-2 {\lambda _2 }) ({\lambda _1 }-{\lambda _2 }+\mu )}. \end{aligned}$$

#### For gapless phases

As in the case of gapped phases, CRG can be performed for gapless phases as well. In our model, curvature function on the critical line $\lambda _2=\mu -\lambda _1$ is

56 $$\begin{aligned} F(k,\mathbf {M})= -\frac{\lambda _1 (\lambda _1-2 \mu )}{2 \left( \lambda _1^2-2 \lambda _1 \mu +2 \mu ^2 + 2 \mu (\mu -\lambda _1) \cos (k) \right) }-1, \end{aligned}$$

here $\mathbf {M}=\left\{ \lambda _1,\mu \right\}$. Second derivative of curvature function at $k_0=0$ can be obtained as

57 $$\begin{aligned} \partial _k^2 F(k,\mathbf {M})|_{k=0}= \frac{{\lambda _1} \mu ({\lambda _1 }-2 \mu ) ({\lambda _1 }-\mu ) \left( {\lambda _1 }^2-2 {\lambda _1 } \mu -2 \mu ({\lambda _1 }-\mu )+2 \mu ^2\right) }{\left( {\lambda _1 }^2-2 {\lambda _1 } \mu +2 \mu (\mu -{\lambda _1 })+2 \mu ^2\right) ^3}. \end{aligned}$$

Derivative of curvature function with respect to the parameters $\lambda _1$ and $\mu$ are correspondingly

58 $$\begin{aligned} \partial _{\lambda _1}F(0,\mathbf {M})&= \frac{\mu }{({\lambda _1 }-2 \mu )^2}, \end{aligned}$$

59 $$\begin{aligned} \partial _{\mu }F(0,\mathbf {M})&= -\frac{{\lambda _1 }}{({\lambda _1 }-2 \mu )^2}. \end{aligned}$$

This gives RG equations for the parameters as

60 $$\begin{aligned} \frac{d\lambda _1}{dl}&= \frac{1}{2}\frac{{\lambda _1} \mu ({\lambda _1 }-2 \mu ) ({\lambda _1 }-\mu ) \left( {\lambda _1 }^2-2 {\lambda _1 } \mu -2 \mu ({\lambda _1 }-\mu )+2 \mu ^2\right) ({\lambda _1 }-2 \mu )^2}{\mu \left( {\lambda _1 }^2-2 {\lambda _1 } \mu +2 \mu (\mu -{\lambda _1 })+2 \mu ^2\right) ^3}\nonumber \\&=-\frac{{\lambda _1} ({\lambda _1 }-\mu )}{2 ({\lambda _1 }-2 \mu )}, \end{aligned}$$

61 $$\begin{aligned} \frac{d\mu }{dl}&= -\frac{1}{2}\frac{{\lambda _1} \mu ({\lambda _1 }-2 \mu ) ({\lambda _1 }-\mu ) \left( {\lambda _1 }^2-2 {\lambda _1 } \mu -2 \mu ({\lambda _1 }-\mu )+2 \mu ^2\right) ({\lambda _1 }-2 \mu )^2}{\lambda _1\left( {\lambda _1 }^2-2 {\lambda _1 } \mu +2 \mu (\mu -{\lambda _1 })+2 \mu ^2\right) ^3}\nonumber \\&=-\frac{\mu (\mu -{\lambda _1})}{2 ({\lambda _1 }-2 \mu )}. \end{aligned}$$

### Derivation of critical exponents

#### For gapped phases

Components of the Hamiltonian, $\chi _{z} (k) = -2 \lambda _1 \cos k - 2 \lambda _2 \cos 2k + 2\mu ,$ and $\chi _{y} (k) = 2 \lambda _1 \sin k + 2 \lambda _2 \sin 2k$, are expanded around HSP $k_0=0$ as

62 $$\begin{aligned} \chi _z&= (2\mu -2\lambda _1-2\lambda _2)+\frac{(8\lambda _2+2\lambda _1)}{2} \delta k^2=\delta g + B \delta k^2, \end{aligned}$$

63 $$\begin{aligned} \chi _y&= (4\lambda _2+2\lambda _1)\delta k =A \delta k \end{aligned}$$

We perform the expansion of $\chi _{z} (k)$ and $\chi _{y} (k)$ for HSP $k_0=\pi$ only upto first order, since the higher order terms are insignificant due to linear spectra around $k_0=\pi$. Thus we have

64 $$\begin{aligned} \chi _z&= (2\mu +2\lambda _1-2\lambda _2)=\delta g , \end{aligned}$$

65 $$\begin{aligned} \chi _y&= (4\lambda _2-2\lambda _1)\delta k=A \delta k \end{aligned}$$

Curvature function for 1D systems can be written in terms of $\chi _{z} (k)$ and $\chi _{y} (k)$ as

66 $$\begin{aligned} F(k,\mathbf {M})= \frac{\chi _y\partial _k \chi _z - \chi _z \partial _k \chi _y}{\chi _z^2+\chi _y^2}. \end{aligned}$$

In the vicinity of HSPs one can write the curvature function in Ornstein-Zernike form in Eq. (9). For HSP $k_0=0$ it reads

67 $$\begin{aligned} \begin{aligned} F(k,\delta g)&= \frac{\left( A\delta k (2B\delta k)-(\delta g+B\delta k^2)A\right) }{\delta g^2 +(2 \delta g B+A^2) \delta k^2 + B^2\delta k^4 }\\&= \frac{\left( \frac{2BA\delta k^2-A(\delta g+B\delta k^2)}{\delta g^2}\right) }{1 + \frac{(2 \delta g B+A^2)}{\delta g^2} \delta k^2 + \frac{B^2}{\delta g^2}\delta k^4 }\\&= \frac{F(k_0,\delta g)}{1+\xi ^2 \delta k^2+\xi ^4\delta k^4}, \end{aligned} \end{aligned}$$

where $F(k_0,\delta g)=\frac{2(\lambda _1+2\lambda _2)}{(2\mu -2\lambda _1-2\lambda _2)} \propto |\delta g|^{-1} \implies \gamma =1$. Correlation length $\xi$ for the transition between $w=0$ and $w=1$ gapped phases is $\xi =\frac{(4\lambda _2+2\lambda _1)}{(2\mu -2\lambda _1-2\lambda _2)}\propto |\delta g|^{-1} \implies \nu =1$, since $\delta k^2$ term dominates over $\delta k^4$. Similarly for the transition between $w=2$ and $w=1$ gapped phases $\xi =\sqrt{\frac{(8\lambda _2+2\lambda _1)}{2(2\mu -2\lambda _1-2\lambda _2)}}\propto |\delta g|^{-\frac{1}{2}} \implies \nu =\frac{1}{2}$, since $\delta k^4$ term dominates over $\delta k^2$.

Following the same procedure in the vicinity of HSP $k_0=\pi$, the curvature function can be written as

68 $$\begin{aligned} \begin{aligned} F(k,\delta g)&= \frac{\left( \frac{A}{\delta g}\right) }{1 + \frac{(A^2)}{\delta g^2} \delta k^2}\\&= \frac{F(k_0,\delta g)}{1+\xi ^2 \delta k^2}, \end{aligned} \end{aligned}$$

where $F(k_0,\delta g)=\frac{2(2\lambda _2-\lambda _1)}{(2\mu +2\lambda _1-2\lambda _2)} \propto |\delta g|^{-1} \implies \gamma =1$. The correlation length $\xi =\frac{(4\lambda _2-2\lambda _1)}{(2\mu +2\lambda _1-2\lambda _2)}\propto |\delta g|^{-1} \implies \nu =1$.

#### For gapless phases

Components of the Hamiltonian expanded around the HSP $k_0=0$, on the critical line $\lambda _2=\mu -\lambda _1$ are,

69 $$\begin{aligned} \chi _z&= 2\mu -2\lambda _1\cos k-2\mu \cos 2k + 2\lambda _1 \cos 2k \end{aligned}$$

70 $$\begin{aligned}&= \left( \frac{8\mu -6\lambda _1}{2}\right) \delta k^2 = B \delta k^2, \end{aligned}$$

71 $$\begin{aligned} \chi _y&= 2\lambda _1\sin k-2\mu \sin 2k + 2\lambda _1 \sin 2k \end{aligned}$$

72 $$\begin{aligned}&=-2(\lambda _1-2\mu )\delta k - \left( \frac{16\mu + 18\lambda _1}{6}\right) \delta k^3 = -2\delta g \delta k- A \delta k^3, \end{aligned}$$

where $(\lambda _1-2\mu )=\delta g$. The curvature function in Ornstein-Zernike form in Eq. (9), can be written as

73 $$\begin{aligned} F(k,\delta g)&=\frac{(-2\delta g \delta k-A\delta k^3)2B\delta k-B\delta k^2(-2\delta t- 3A \delta k^2)}{(B\delta k^2)^2 +(-2\delta g \delta k-A\delta k^3)^2} \end{aligned}$$

74 $$\begin{aligned}&= \frac{-2B\delta g \delta k^2 + AB \delta k^4}{4 \delta g^2 \delta k^2 + (B^2+4\delta g A) \delta k^4 + A^2 \delta k^6} \end{aligned}$$

75 $$\begin{aligned}&= \frac{\left( \frac{-2B\delta g \delta k^2 + B A \delta k^4}{4 \delta g^2 \delta k^2}\right) }{1+\left( \frac{A^2+4\delta g B}{4 \delta g^2}\right) \delta k^2 + \left( \frac{B^2}{4 \delta g^2} \right) \delta k^4 } \end{aligned}$$

76 $$\begin{aligned}&=\frac{F(k_0,\delta g)}{1+\xi ^2\delta k^2+\xi ^4 \delta k^4}, \end{aligned}$$

where $F(k_0,\delta g)=\frac{(4\mu -3\lambda _1)}{2(\lambda _1-2\mu )} \propto |\delta g|^{-1} \implies \gamma =1$. The correlation length $\xi =\sqrt{\frac{8\mu -6\lambda _1}{4(\lambda _1-2\mu )}}\propto |\delta g|^{-\frac{1}{2}} \implies \nu =\frac{1}{2}$, since $\delta k^4$ term is dominant.

### Derivation of modified scaling law

In order to preserve the constant value of topological invariant, the divergence of the curvature function near HSP, as we approach the transition point ($\mathbf {M}\rightarrow \mathbf {M}_{\mathbf{c}}$), has to be conserved^28^. The contribution to the topological invariant from the divergence $C_{div}$ of curvature function near the HSP $k_0=0$, as we approach CP-2, can be obtained by integrating over the width $\xi ^{-1}$

77 $$\begin{aligned} C_{div} =F(k_0,\delta g) \int \limits _{-\xi ^{-1}}^{\xi ^{-1}} \frac{d\delta k}{(1+\xi ^4\delta k^4)}, \end{aligned}$$

here

78 $$\begin{aligned} \int \limits _{-\xi ^{-1}}^{\xi ^{-1}} \frac{d\delta k}{(1+\xi ^4\delta k^4)}&= \frac{1}{\sqrt{\xi ^4}}\tan ^{-1}\left( \sqrt{\xi ^4} \delta k^2 \right) |_{-\xi ^{-1}}^{\xi ^{-1}} \end{aligned}$$

79 $$\begin{aligned}&= \frac{1}{\xi ^2}\left( \tan ^{-1}(-1) - \tan ^{-1}(1)\right) \end{aligned}$$

80 $$\begin{aligned}&=\frac{1}{\xi ^2} \left( \frac{\pi }{2}\right) \end{aligned}$$

Thus we have

81 $$\begin{aligned} C_{div} =\frac{F(k_0,\delta g)}{\xi ^2} \times \mathbf {O}(1) = \text {constant}. \end{aligned}$$

Combining this with Eq. (13) (i.e, $F(k_0,\mathbf {M}) \propto |\mathbf {M}-\mathbf {M}_c|^{-\gamma } ,\;\;\; \xi \propto |\mathbf {M}-\mathbf {M}_c|^{-\nu }$), we get the modified scaling law for 1D as

82 $$\begin{aligned} \gamma =2\nu . \end{aligned}$$

### Calculations of correlation function

The critical line $\lambda _2=\mu -\lambda _1$ which occurs at $k_0=0$, has distinct gapless phases, CP-1 and CP-2. As we approach the CP-1, the correlation function $\lambda _R$ can be obtained as

83 $$\begin{aligned} \lambda _R&= \int _{-\infty }^{\infty } \frac{dk}{2\pi } e^{ikR}F(k,\mathbf {M}) \end{aligned}$$

84 $$\begin{aligned}&= \int _{-\infty }^{\infty } \frac{dk}{2\pi } \frac{F(0,\mathbf {M})}{1+\xi ^2k^2} e^{ikR} \end{aligned}$$

85 $$\begin{aligned}&= \frac{F(0,\mathbf {M})}{2\xi } e^{-|R|/\xi } \end{aligned}$$

In terms of the parameters of the model Hamiltonian the above equation reads

86 $$\begin{aligned} \lambda _R=\frac{1}{2\xi } \left( \frac{2 (\lambda _1+2 \lambda _2)}{2 \mu -2 \lambda _1-2 \lambda _2}\right) \exp \left( -\frac{|R|}{\xi }\right) \end{aligned}$$

where $\xi =\frac{2 (\lambda _1+2 \lambda _2)}{2 \mu -2 \lambda _1-2 \lambda _2}$. Similarly as we approach the CP-2, $\lambda _R$ can be obtained as

87 $$\begin{aligned} \lambda _R&= \int _{-\infty }^{\infty } \frac{dk}{2\pi } e^{ikR}F(k,\mathbf {M}) \end{aligned}$$

88 $$\begin{aligned}&= \int _{-\infty }^{\infty } \frac{dk}{2\pi } \frac{F(0,\mathbf {M})}{1+\xi ^4k^4} e^{ikR} \end{aligned}$$

89 $$\begin{aligned}&= \frac{F(0,\mathbf {M})}{2\sqrt{2}\xi } \left( \cos \left[ \frac{|R|}{\sqrt{2}\xi }\right] +\sin \left[ \frac{|R|}{\sqrt{2}\xi }\right] \right) e^{-|R|/\sqrt{2}\xi }. \end{aligned}$$

In terms of parameters of the model Hamiltonian it reads

90 $$\begin{aligned} \lambda _R=\frac{1}{2\;\xi \sqrt{2}}\left( \frac{2 (\lambda _1+2 \lambda _2)}{2 \mu -2 \lambda _1-2 \lambda _2}\right) \left\{ \sin \left( \frac{\left| R\right| }{\sqrt{2}\;\xi }\right) +\cos \left( \frac{\left| R\right| }{\sqrt{2}\;\xi }\right) \right\} \exp {\left( - \frac{|R|}{\sqrt{2} \;\xi }\right) } \end{aligned}$$

where $\xi = \sqrt{\frac{2 \lambda _1+8 \lambda _2}{2 (2 \mu -2\lambda _1-2\lambda _2)}}$. For the critical line $\lambda _2=\mu +\lambda _1$ which occurs at $k_0=\pi$, the $\lambda _R$ can be obtained as

91 $$\begin{aligned} \lambda _R&= \int _{-\infty }^{\infty } \frac{dk}{2\pi } e^{ikR}F(k,\mathbf {M}) \end{aligned}$$

92 $$\begin{aligned}&= \int _{-\infty }^{\infty } \frac{dk}{2\pi } \frac{F(\pi ,\mathbf {M})}{1+\xi ^2k^2} e^{i(\pi +k)R} \end{aligned}$$

93 $$\begin{aligned}&= F(\pi ,\mathbf {M})\frac{e^{\left( i\pi R-\frac{R}{\xi }\right) }}{2\xi } \end{aligned}$$

94 $$\begin{aligned}&= (-1)^R\frac{F(\pi ,\mathbf {M})}{2\xi } e^{-|R|/\xi } \end{aligned}$$

Since the bulk gap closes at $k_0=\pi$ the sign alternates between even and odd sites. In terms of the parameters of the model Hamiltonian the above equation reads

95 $$\begin{aligned} \lambda _R=\frac{(-1)^R}{2\;\xi }\left( \frac{2 (2 \lambda _2 -\lambda _1)}{2 \lambda _1-2 \lambda _2 +2 \mu }\right) \exp \left( -\frac{|R|}{\xi }\right) , \end{aligned}$$

where $$\xi =\left( \frac{4 \lambda _2-2 \lambda _1}{2 \mu +2 \lambda _1 -2 \lambda _2 }\right) $$.
